# Enhancing Interoperability for a Sustainable, Patient-Centric Health Care Value Chain: Systematic Review for Taxonomy Development

**DOI:** 10.2196/69465

**Published:** 2025-04-25

**Authors:** Carlos Antonio Marino, Claudia Diaz Paz

**Affiliations:** 1 CENTRUM Católica Graduate Business School, Lima, Peru Pontifical Catholic University of Peru Lima Peru

**Keywords:** interoperability, health care, electronic health record (EHR), Fast Healthcare Interoperability Resources (FHIR), value chain

## Abstract

**Background:**

Creating a sustainable, patient-centered health care system necessitates integrated supply chains supported by information technologies. However, achieving interoperability among various devices and systems remains a significant hurdle. Our research highlights the need for systematic reviews that address health care interoperability as a holistic knowledge domain. Notably, we observed a lack of studies that outline its structure or develop a comprehensive, high-order facet-based taxonomy from the perspective of supply or value chains. This study aims to address that gap.

**Objective:**

The primary aim of this study is to elucidate the knowledge structure within the extensive domain of health care interoperability, with an emphasis on trending topics, critical hot spots, and the categorization of significant issues. Furthermore, we aim to model the higher-order elements of a taxonomy for health care interoperability within the context of the health care value chain framework.

**Methods:**

We used both quantitative and qualitative methodologies. The PRISMA (Preferred Reporting Items for Systematic Reviews and Meta-Analyses) framework guided our selection process. We examined 6 databases—Scopus, Web of Science, IEEE Xplore, Embase, Cochrane, and PubMed—focusing on journal articles and gray literature published from 2011 onward. Articles were screened using predefined eligibility criteria. Quantitative bibliometric techniques—including cluster, factor, and network analyses—were applied to explore the structure of the knowledge. A subset of articles was selected for qualitative synthesis using an iterative coding process to develop a higher-order facet-based taxonomy.

**Results:**

We identified 370 articles for quantitative analysis. The bibliometric analysis revealed 2 major clusters. Key terms in the first cluster included interoperability, electronic health record, and eHealth—with betweenness centralities of 70.971, 59.460, and 12.000, respectively, and closeness centralities of 0.047, 0.043, and 0.034, respectively. In the second cluster, the most relevant terms were IoT, blockchain, and health care—with betweenness centralities of 6.765, 2.581, and 1.283, respectively, and closeness centralities of 0.034, 0.030, and 0.030, respectively. Factor analysis explained 59.46% of the variance in a 2-factor model, with the first dimension accounting for 36.78% and the second dimension for 22.68%. The qualitative review of 79 articles yielded a taxonomy with 4 higher-order facets: object (what is shared), source (what mechanism is used), ambit (space covered), and content (technology primarily involved). Each facet extended to a third level of classification.

**Conclusions:**

The comprehensive domain of health care interoperability, viewed through the lens of a sustainable value chain, encompasses studies that highlight various facets or attributes. These studies underscore the relevance of eHealth within this knowledge domain and reflect a strong focus on 2 key health information technologies: electronic health records and the Internet of Things.

## Introduction

### Background and Motivation

Patient centricity—a health care approach that prioritizes patients’ needs, preferences, and experiences, ensuring they are active participants in their own care and decision-making—has emerged as a core principle in contemporary health care practices [1-4], closely linked to the ideas of sustainability and responsiveness [4]. Empirical evidence supports the importance of an integrated supply chain in enhancing patient centricity [5]. Studies have shown a direct and positive relationship between integrated supply chain performance and patient centricity, as well as between knowledge exchange and integrated supply chain performance. In this context, information technologies play a crucial role [1,4,6], while achieving interoperability remains a challenge that must be addressed to improve supply chain integration.

Three main concepts are linked to patient centricity and advances in digital innovation: (1) service inclusivity, (2) shared responsibility or management, and (3) remote patient monitoring [4]. Moreover, these concepts are reflected in 3 prominent roles: facilitator, connector, and enabler [4]. The first provides patients with access to services and data, the second enables connection and information sharing across health care infrastructure, and the last pertains to remote health care [4]. In this context, interoperable health care systems—when viewed through the lens of the supply chain or value chain—play a critical role in advancing patient centricity. This perspective emphasizes the need for seamless integration and data exchange among stakeholders across the health care ecosystem, thereby supporting holistic patient care and promoting a more integrated approach to health delivery. The alignment of these systems with patient-centric principles highlights the importance of collaboration, efficiency, and improved health outcomes in the evolving landscape of health care delivery.

The relevance of interoperability for a sustainable, patient-centric health care value chain requires a holistic understanding of the domain—one that also addresses the ongoing debate surrounding the classification of interoperability. Some authors propose simple classifications comprising 2 elements—syntactic and semantic interoperability [7]—or 3 elements—technical, syntactic, and semantic [8]. Other studies suggest as many as 8 [9] or even 12 classes [10]. References can be found to various types of interoperability, including technical [9,11], syntactic or syntactical [9,11], pragmatic [9,11], dynamic [9], conceptual [9,11], structural [9], functional [9], semantic [9,11], platform [11], process [12], organizational [13], people [14], knowledge [10], services [10], social networks [10], electronic identity [10], ecosystem [10], and legal interoperability [15,16].

Previous reviews on interoperability have not focused on specific industries such as health care, nor have they consistently provided structured classifications. One review addressed user model interoperability but lacked a systematic approach [17]. Another study examined evaluation models of interoperability, categorizing 12 models across 4 granularity levels [10]. While this study mentioned interoperability attributes, these were not developed through a systematic procedure [10]. Additionally, an investigation explored interoperability in the context of the Internet of Things, presenting a comprehensive taxonomy [11]. Nevertheless, the proposed taxonomy is based on a predefined model rather than a specific methodology for taxonomy development [11]. Furthermore, it is not a facet-based taxonomy.

There are also reviews focused on interoperability in health care. Nevertheless, most of them lack a comprehensive scope within the domain. They are often limited to a specific health care service, such as emergency care [18]; a particular technology, such as blockchain [19]; a type of health information technology (HIT), such as electronic health records (EHRs) [20,21]; or a specific type of interoperability, such as semantic interoperability [16,21-24]. One of these surveys proposed a taxonomy for the semantic interoperability of health record systems; however, its development did not follow a specific methodology [21], and the taxonomy was not facet based. Similarly, we found reviews focused on specific and relevant health interoperability standards or technologies [25-29], yet none of them included a taxonomy.

Three studies addressed the topic of interoperability in health care using a systematic and broader approach compared with previous studies [9,30]. The first was a literature review that assessed 61 records [30], focusing on identifying functional requirements for data integration. In line with its scope, this study did not include a quantitative field assessment. The second study surveyed 36 articles to present the interoperability requirements for health information systems [9]. This survey presented word clouds and independently classified and summarized interoperability standards and architecture components, all grounded in the literature review [9]. However, it did not offer a comprehensive organization of the knowledge domain, as the classifications were not integrated. Notably, the study did not propose a taxonomy, nor did it follow a specific methodology for taxonomy development or identify the facets of the topic. The third study conducted a bibliometric analysis of system interoperability and data linkage in health care, including both cluster and network analyses of the topic [31]. The authors selected a sample of 63 journal articles based on their average citations per year, among other criteria [31]. While this criterion is relevant, it may exclude more recent articles that have not yet accumulated sufficient citations, as well as gray literature—only 1 conference paper was included in the sample [31]. In line with its approach, the study did not propose a taxonomy grounded in a literature review. Furthermore, no studies were found that examined interoperability in health care from a supply chain or value chain perspective.

Following a review of the relevant studies, we identified a clear scarcity of systematic reviews that address interoperability in health care as a comprehensive knowledge domain. There is a need for a thematic structure and a higher-order, facet-based taxonomy of knowledge, particularly from the perspective of the supply or value chain. This study aims to fill that gap.

A systematic literature review has been defined as a type of research synthesis [32] that employs predefined methods aimed at reducing bias, thereby producing more reliable results [32]. These reviews incorporate various methods of data analysis [33], including quantitative approaches such as bibliometrics [34], as well as qualitative, in-depth document analysis. Additionally, the literature suggests systematic methods for taxonomy development [35,36]. Consequently, a systematic approach is the most appropriate for our inquiry.

A holistic perspective—currently lacking in the literature and pursued in this study—helps eliminate knowledge silos and can be achieved through the integration of both quantitative and qualitative methodologies. The use of machine learning tools enables the handling of large volumes of information, offering critical insights into the knowledge structure of this comprehensive domain. Additionally, a thorough review of a selected sample [31,37] supported the development of a facet-based taxonomy that captures the higher-order facets of the topic. Previous studies in other subject areas have used systematic approaches, combining quantitative bibliometric assessments with taxonomies derived from in-depth literature reviews [38].

### Objectives and Research Questions

This research pursues 2 fundamental objectives. First, it seeks to vividly map the intricate knowledge structure of the expansive field of health care interoperability, viewed through the compelling lens of the value chain. This exploration highlights not only the critical topics and emerging hot spots that shape the discourse, but also the complex interconnections that bind key themes together. Second, it aims to develop a robust, higher-order framework for a taxonomy of interoperability in health care, offering a nuanced perspective that underscores its significance within the evolving landscape of the health care value chain.

## Methods

### Search Strategy

This review aims to be both comprehensive and grounded in high-quality data. To achieve this, the initial search was designed to capture a broad spectrum of information, including both peer-reviewed articles and gray literature. However, to ensure consistency and minimize subjective judgment regarding data quality and selection bias, we chose to rely on specialized databases that index only peer-reviewed documents.

Data were retrieved from 6 databases on April 1, 2024, in accordance with PRISMA (Preferred Reporting Items for Systematic Reviews and Meta-Analyses) and PRISMA-S (Preferred Reporting Items for Systematic reviews and Meta-Analyses—Search extension) guidelines (see Multimedia Appendix 1) [39,40]. The databases consulted were (1) Scopus, (2) Web of Science (WoS), (3) IEEE Xplore, (4) Embase, (5) Cochrane, and (6) PubMed. In Scopus, the search was limited to the Title-Abstract-Keywords fields; in WoS, it was restricted to the Topic field; and in Cochrane, it was confined to the Title, Abstract, and Keyword fields. These limitations were applied to avoid the inclusion of irrelevant records, as broader fields—such as References in Scopus—could yield inaccurate results. For IEEE Xplore, Embase, and PubMed, no field restrictions were applied, ensuring the most comprehensive coverage of relevant literature.

The search terms focused on key themes such as interoperability, value chains, and supply chains within the context of health care. Notably, multiword terms were searched as exact phrases to prevent inaccuracies that might result from search systems interpreting the words separately (Textbox 1).

**Search terms.**

1. Interoperability: (interoperability OR inter-operability OR “inter operability” OR “data structure” OR data-structure OR “data standard” OR data-standard)
2. Health care supply chain: (“health care value chain” OR “health care supply chain” OR e-health OR “health care value-chain” OR “health care supply-chain” OR “health care supplychain”).
3. The connector between both concepts: AND

The eligibility criteria for document types (articles or conference papers) were aligned with the structure of each database, aiming to maintain consistency across sources as much as possible. The criteria were as follows: (1) conference paper and article for Scopus; (2) article and proceeding paper for WoS; (3) conferences and journals for IEEE Xplore; (4) article and conference paper for Embase; and (5) classical article, clinical conference, consensus development conference, consensus development conference (NIH), and corrected and republished articles for PubMed. Additionally, the review was limited to English-language materials to reduce potential bias in the quantitative analysis. These restrictions were applied using the databases’ built-in filters where available.

The period for the review was set from 2011 to 2024, considering that one of the most significant standards in health care—Fast Healthcare Interoperability Resources (FHIR)—was introduced in 2011 [27]. The eligibility criterion regarding topical relevance—whether the document focused on the subject under study—required subjective evaluation by the researchers. In line with recommendations from the literature, which emphasize the importance of a peer-review stage to reduce selection bias [39], this evaluation was conducted in 2 stages and involved both researchers. Following the approach adopted in previous studies [41], one of the researchers (CPD) conducted a thorough review of titles and abstracts to perform the initial selection. This selection was then peer reviewed by the second researcher (CAM). Any doubts or discrepancies were discussed and resolved collaboratively. As this process relied on the review of titles and abstracts, it was decided in advance to exclude any articles lacking abstracts. Microsoft Excel was used to manage and document the screening process. The eligibility criteria are summarized in Textbox 2.

**Eligibility criteria.**

**1. Inclusion criteria**

- Article or conference paper
- Focused on interoperability in health care
- English language
- Published in 2011 or later

**2. Exclusion criteria**

- Editorial, summary, news, or technical discussion
- Not focused on interoperability in health care
- Non-English language
- Published before 2011

The data retrieved from the various databases were first merged, cleaned, and prepared for analysis. The data cleaning process involved removing duplicates [42], correcting formatting issues [43], and addressing other errors such as spelling inconsistencies [44]. The literature outlines a variety of approaches for this stage. Some studies have conducted manual data reviews [45], acknowledging the effort required [46]. Others have utilized tools such as Microsoft Excel for data cleaning [45,47], while additional research has developed or used specific software tools—including custom packages [46,48]—or dedicated procedures [49]. Notably, some authors have reported that even after using software tools, additional manual data cleaning was necessary and subsequently performed [48,50]. Based on this, we opted to carry out a manual process using Microsoft Excel for data merging and cleaning. Furthermore, the literature presents differing views on the criteria for identifying duplicate records. Some studies rely solely on digital object identifiers (DOIs) [46], while others combine DOIs with additional attributes [46], or use alternative attributes entirely [51]. Considering these options, we chose to identify duplicates based on DOIs, as this is regarded as the most reliable method.

The procedure we followed began with retrieving and exporting bibliometric data from multiple databases. The data were then merged according to the structure of the Scopus database. Subsequently, we conducted data cleaning based on the 2-step approach recommended in the literature [44]. The first step involved detecting and removing duplicates, while the second focused on identifying and correcting errors and inconsistencies in preparation for bibliometric analysis [44]. Initially, records without a DOI were excluded. Duplicate records were identified based on duplicated DOIs, with only 1 instance of each retained. In the second step, error correction was carried out. This phase can be conducted using either Microsoft Excel or a thesaurus-based approach [44]; we applied both methods to ensure accuracy and consistency. First, we used Microsoft Excel to correct special characters in the columns identified as relevant for our study, as flagged by Biblioshiny (K-Synth Srl). Second, we applied a thesaurus-based approach during the keyword co-occurrence analysis to standardize terminology. Specifically, we treated the following terms as synonyms: eHealth and e-health; electronic health record, electronic health records, and EHR; IoT and Internet of Things; IoMT and Internet of Medical Things.

Missing data were also identified and addressed during the data preparation process. Certain statistical techniques commonly used in bibliometric studies—such as multiple correspondence analysis and network analysis—require complete data sets to avoid potential bias [52,53]. Therefore, it was essential to assess the extent of missing data [54]. According to the literature, missing data rates of 10% or less are generally considered low [54], and in such cases, any imputation strategy may be applied. One commonly used approach is complete-case analysis, which involves deleting records with missing data.

### Data Analysis and Synthesis

The data synthesis process consists of 2 stages: quantitative and qualitative. The literature supports the use of both quantitative and qualitative techniques for the development of classifications [36]. A key component of bibliometric analysis is the identification of the most relevant themes or topics within the domain under study [55,56]. Accordingly, the first stage involves bibliometric evaluation techniques, including the application of unsupervised machine learning methods. These are used to conduct network analysis, clustering, and multiple correspondence analysis to explore influential topics, emerging hot spots, and the interrelationships among key issues and thematic groups. This approach aims to reveal the knowledge structure of the field from a holistic perspective.

The primary quantitative technique used to reveal the knowledge structure of the domain was a co-occurrence analysis based on keyword terms. This method is particularly well-suited for identifying hot topics and thematic areas [57], and it provides insights into the most frequently discussed subjects within the field [38]. As previously mentioned, the study drew data from several databases, some of which include enriched keywords generated by proprietary editorial algorithms. To minimize potential bias introduced by varying editorial criteria or algorithms, we chose to exclude these enriched keywords and relied solely on those provided by the authors. The data assessment was conducted using Biblioshiny, the web interface of the Bibliometrix R package (version 4.1; R Foundation), which provided the tools necessary for performing the bibliometric analysis.

Cluster and factor analyses are techniques commonly used to classify elements within a data set. In accordance with the literature on classification methods [35], which defines the objective as “grouping objects of interest in a domain based on common characteristics,” clusters consisting of only a single element were excluded, as they do not meet this criterion. The cluster analysis was conducted using the Walktrap algorithm. We selected this algorithm because it is one of the most utilized in community detection [58] and “it is often effective at determining the correct number of communities and assigning items to their proper community” [58]. Other studies also acknowledge Walktrap as a well-performed algorithm [59]. In addition, we decided to perform the analysis based on other algorithms to avoid possible algorithmic bias. The Leading Eigenvalues algorithm, which also has a good performance in community detection [60], was selected.

After the cluster analysis, we performed the factor analysis. It is relevant to mention that the literature emphasizes that the distinction between clustering techniques and factor analysis lies in the way variance is partitioned [61]. In factor analysis, variance is distributed among factors, and the elements have loadings on the different factors within the solution [61]. Thus, we considered it pertinent to present and compare both solutions—those from the cluster and factor analyses.

The COVID-19 pandemic posed a significant challenge to the health care sector. Consequently, we examined the thematic evolution of this field, identifying the year 2020 as a critical turning point and using it as the final stage of the quantitative analysis.

The second stage was qualitative and involved a thorough analysis of the most relevant documents to develop a facet-based taxonomy. A key characteristic of such taxonomies is that they incorporate multiple perspectives that describe a topic [62] and, once completed, enable the use of compound terms—drawn from the same facet or across multiple facets—to define the object of interest [62]. As the meta-characteristic of this taxonomy [35,36], we established that aspects of interoperability would be integrated under the perspective of the health care value chain. We adopted an inductive [35], empirical-to-conceptual approach [36,61], based on an in-depth review of a selected sample of documents. This approach is most suitable when the objective is descriptive, as is the case in our study [36]. The process aimed to formulate a label or concept representing the type [61] and, in line with the literature, followed 3 stages [36] applied to the selected sample. First, each document was read in detail and summarized. Basic and recurrent topics—or codes—were identified and listed. Second, the common characteristics of these topics were identified, and the topics were grouped into categories based on their shared attributes. It was noted that some categories needed to be included within others at a higher level. This second step enabled the identification of the third- and second-level categories, or subfacets. Third, the second-level subfacets were reviewed and organized into higher-level dimensions or facets, representing the first-level facets. One of the researchers (CPD) carried out these steps and formulated an initial proposal. This proposal was then peer reviewed and evaluated by the other researcher (CAM). Any disagreements were discussed and resolved jointly by both researchers (CPD and CAM) until a final version was reached.

In addition, we followed the iterative process suggested by the literature [35,36] and evaluated the fulfillment of ending conditions [35]. The ending conditions we established were (1) having a representative and balanced sample composed of both journal and conference articles, with journal articles forming the majority to ensure the quality of the taxonomy; and (2) achieving category saturation—meaning no additional insights were found [63] regarding the categories under study—given the qualitative nature of this analysis.

Previous studies that conducted bibliometric evaluations selected a sample of studies for an in-depth review that follows and complements the quantitative analysis [37]. To reduce potential bias stemming from researcher subjectivity in the sample selection, this study based the sample selection primarily on the contribution of articles to the factorial solution obtained in the quantitative assessment.

## Results

### Overview

**Figure 1:**
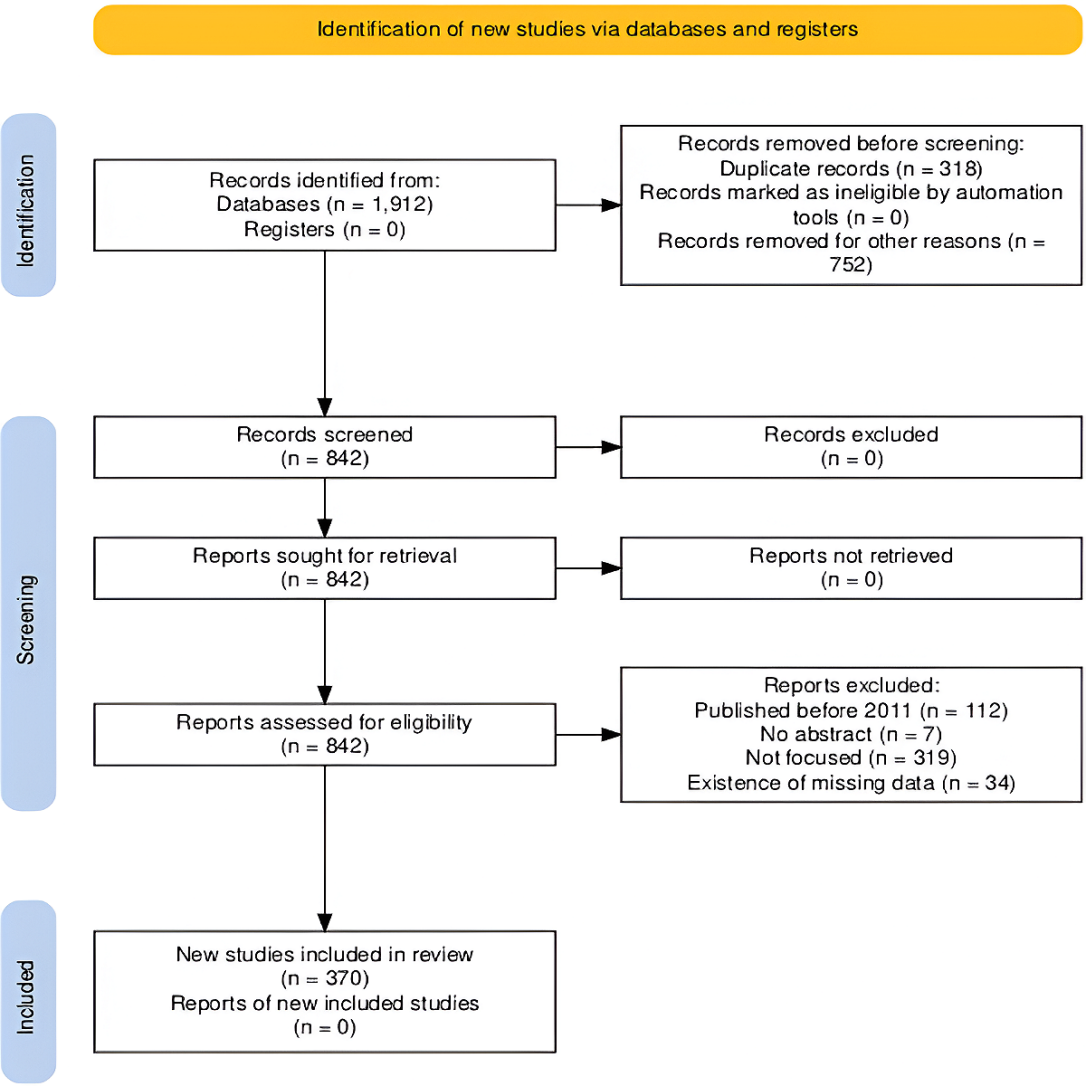
Data identification based on the PRISMA (Preferred Reporting Items for Systematic Reviews and Meta-Analyses) framework.

The search yielded 1912 documents (Scopus: 610; WoS: 161; IEEE Xplore: 303; PubMed: 191; Embase: 643; and Cochrane: 4). Among these, 540 were excluded because they were not articles or conference papers or were not written in English. Additionally, 212 documents lacked a DOI, and 318 were duplicates; these were also removed. At the end of the identification stage, 842 documents remained for screening. It was found that 112 records were published before 2011, 7 did not include abstracts, and 319 were not focused on the topic. These documents were also excluded. Additionally, 34 out of 404 (8.4%) documents lacked authors’ keywords and were withdrawn, as we applied the complete data imputation strategy. Ultimately, 370 records remained. The list of these 370 studies is available on GitHub (see Multimedia Appendix 2). The stages of document identification, screening, and inclusion are illustrated in Figure 1 (also see Multimedia Appendix 1).

### Descriptive Statistics

**Figure 2:**
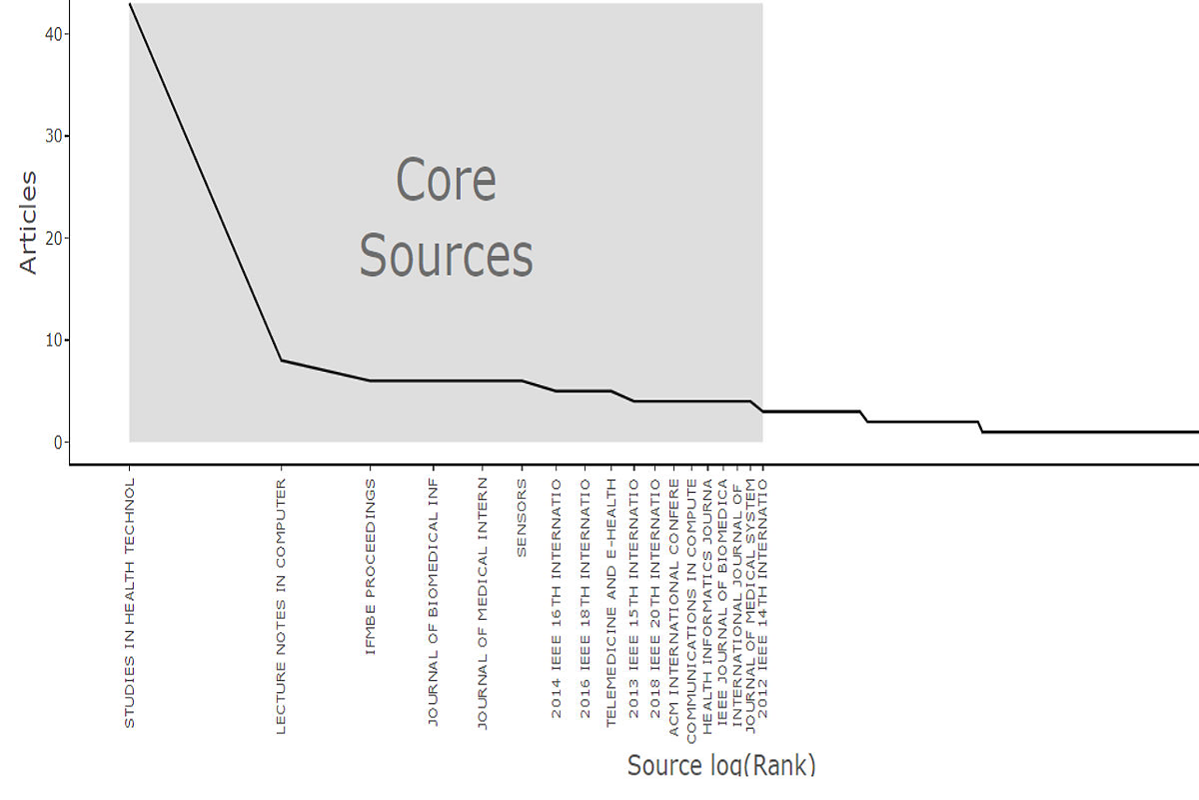
Most relevant journals that published articles regarding interoperability in health care.

The data spans 370 documents published between 2011 and 2024, distributed across 223 sources. The most relevant source is Studies in Health Technology and Informatics, and 48 sources published 2 or more documents. Figure 2 presents the core sources based on Bradford’s Law. Bradford’s Law is a bibliometric distribution measure [64] that reflects journal productivity [64,65], based on the number of accumulated articles [65]. In this regard, it is worth noting that 17 sources comprise the nucleus or core sources. Nevertheless, Studies in Health Technology and Informatics is clearly the most productive one.

**Figure 3:**
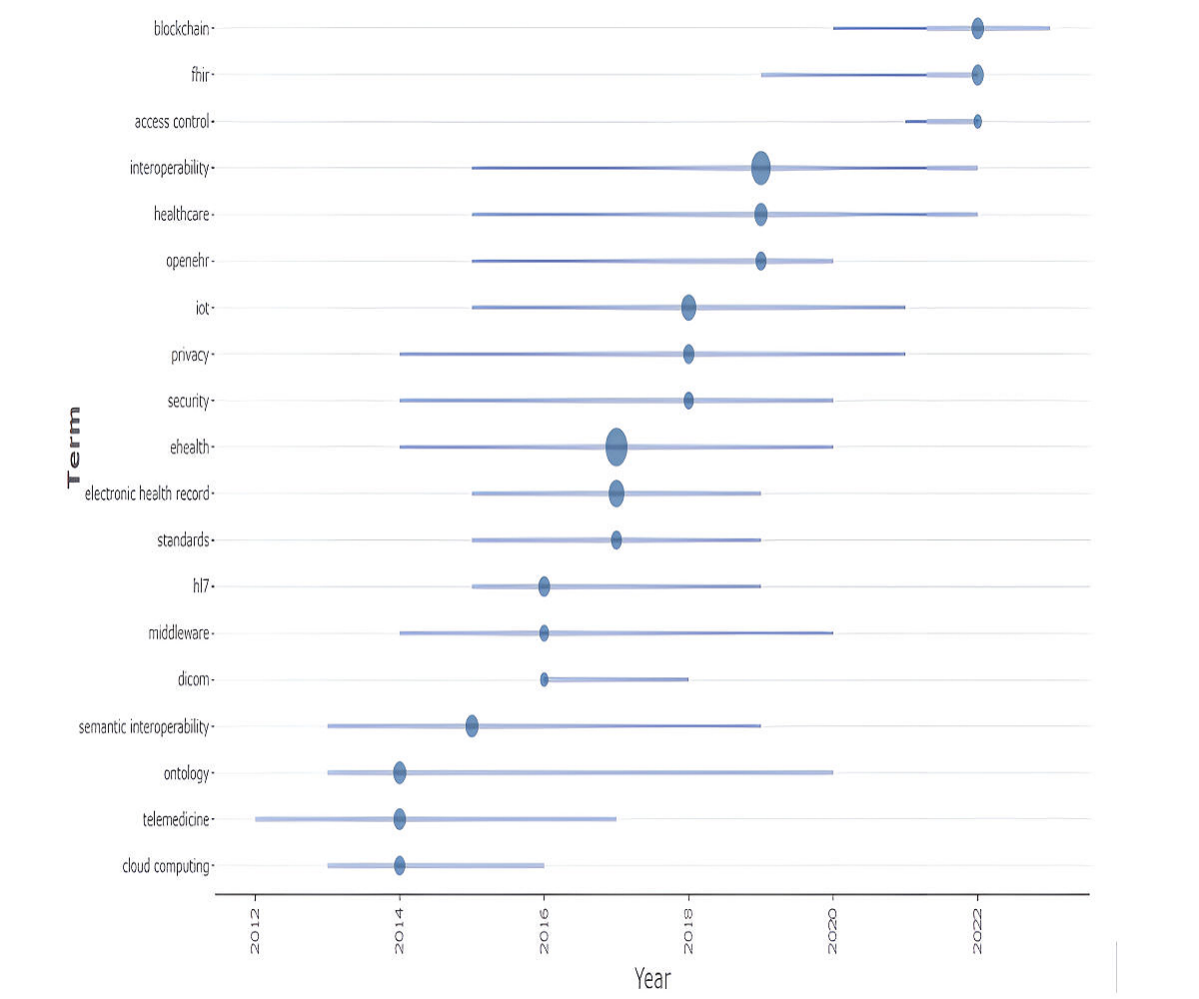
Trend topics in interoperability in health care. FHIR: Fast Healthcare Interoperability Resources; HL7: Health Level Seven; IoT: Internet of Things.

In addition, 358 out of the 370 (96.8%) documents were coauthored, with an average of 5.38 researchers per study. A total of 1008 authors’ keywords were identified. The trend topic analysis encompassed 19 concepts. Among them, ontology, telemedicine, and cloud computing had the oldest mean year of use, while blockchain, FHIR, and access control had the most recent (see Figure 3). This pattern reflects the growing relevance of new technologies, such as distributed ledger technology (blockchain), in this field. It is not surprising that access control also gained relevance in recent years, given that blockchain, due to its immutability and traceability, promises to enhance privacy and security in access management. Additionally, it is noteworthy that the FHIR standard gained prominence only after 2018, despite being issued in 2011 [27]. This may reflect the time required for a new standard to be widely disseminated. The average annual scientific production, excluding the year 2024, is 27.76.

### Bibliometric Analysis

**Figure 4:**
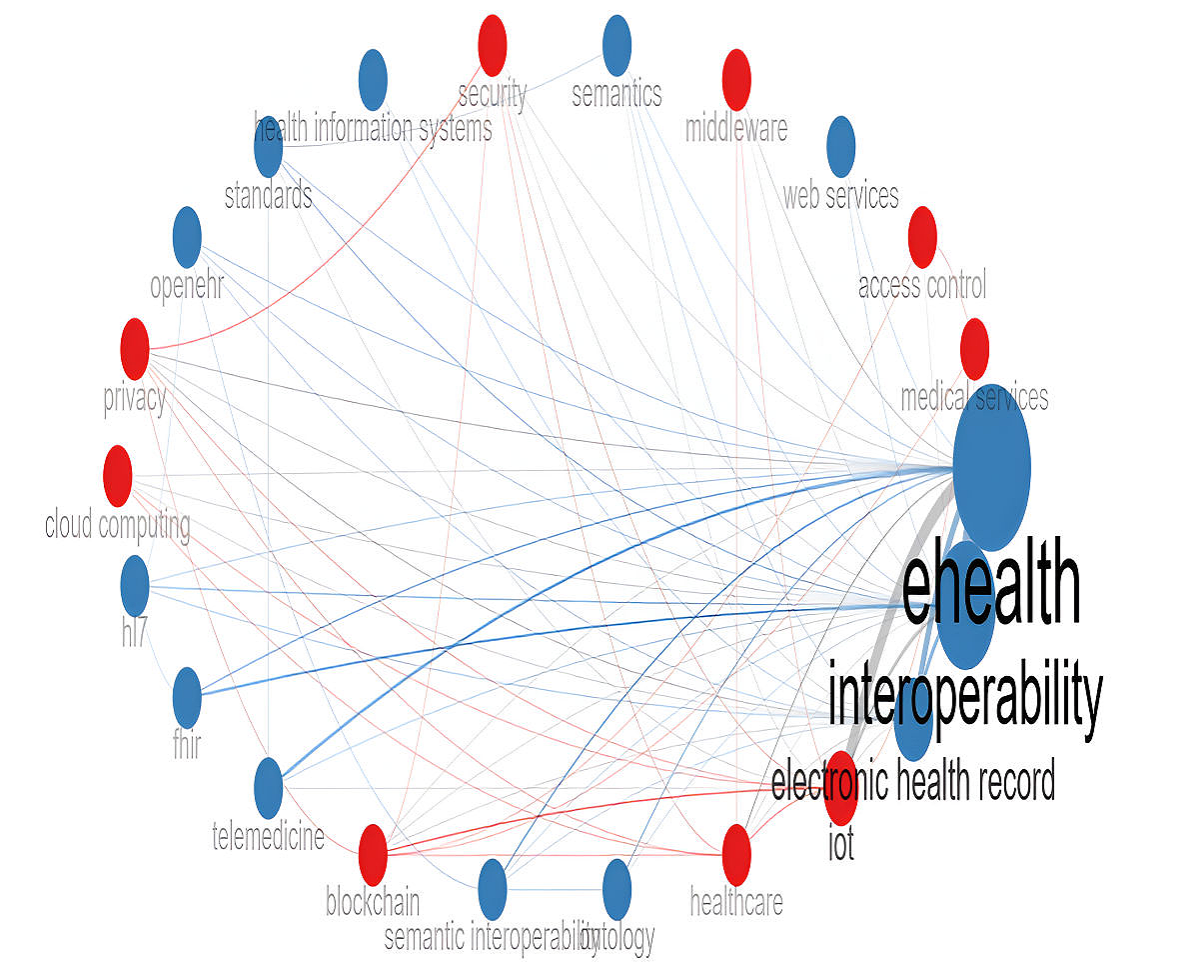
Network and clusters of themes of interoperability in health care.

We performed the cluster analysis using 2 algorithms to avoid algorithmic bias: (1) Walktrap—as the main one—and (2) Leading Eigenvalues—as the confirmatory one. Both solutions resulted in a 2-cluster configuration based on 23 nodes. Figure 4 depicts the solution generated by the Walktrap algorithm. The number of members and the specific elements within each cluster were almost identical in both algorithmic solutions. The only difference was the placement of the term EHR, which appeared in different clusters depending on the algorithm used.

The first cluster encompassed the terms eHealth, interoperability, EHR, ontology, semantic interoperability, telemedicine, FHIR, Health Level Seven (HL7), OpenEHR, standards, health information, system, semantics, and web services. The second cluster included the terms IoT, health care, blockchain, cloud computing, privacy, security, middleware, access control, and medical services. The network analysis revealed that 3 terms had the highest influence on node communication within the entire network. These are interoperability, EHR, and eHealth, with betweenness centrality scores of 70.971, 59.460, and 12.000, respectively. These terms also contribute to the speed of information dissemination within the network, showing the highest closeness centrality scores: 0.047, 0.043, and 0.034, respectively. All 3 terms are grouped within the first cluster. In the second cluster, the terms IoT, blockchain, and health care exhibited the highest levels of betweenness centrality—6.765, 2.581, and 1.283, respectively—as well as the highest closeness centrality scores: 0.034, 0.030, and 0.030, respectively.

**Figure 5:**
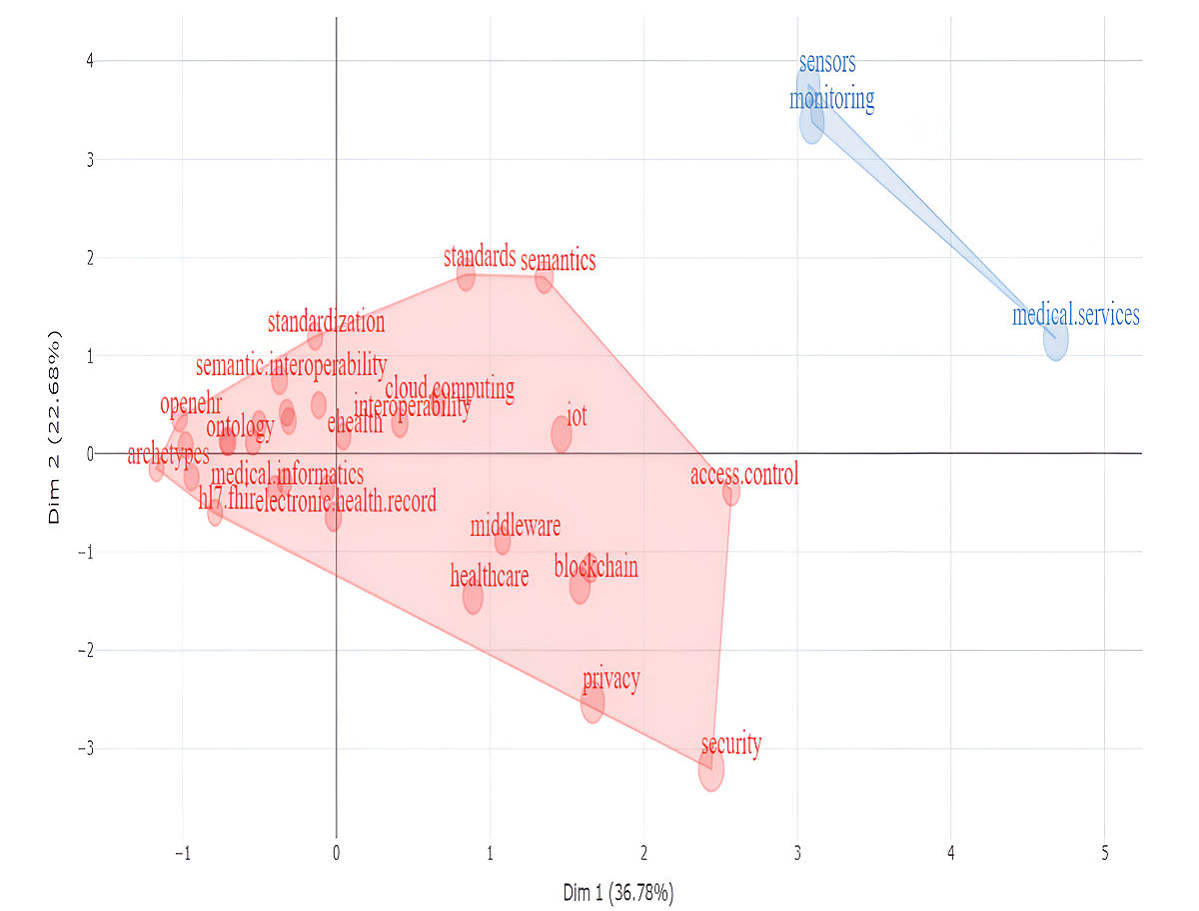
Factors formed by themes of interoperability in health care.

**Figure 6:**
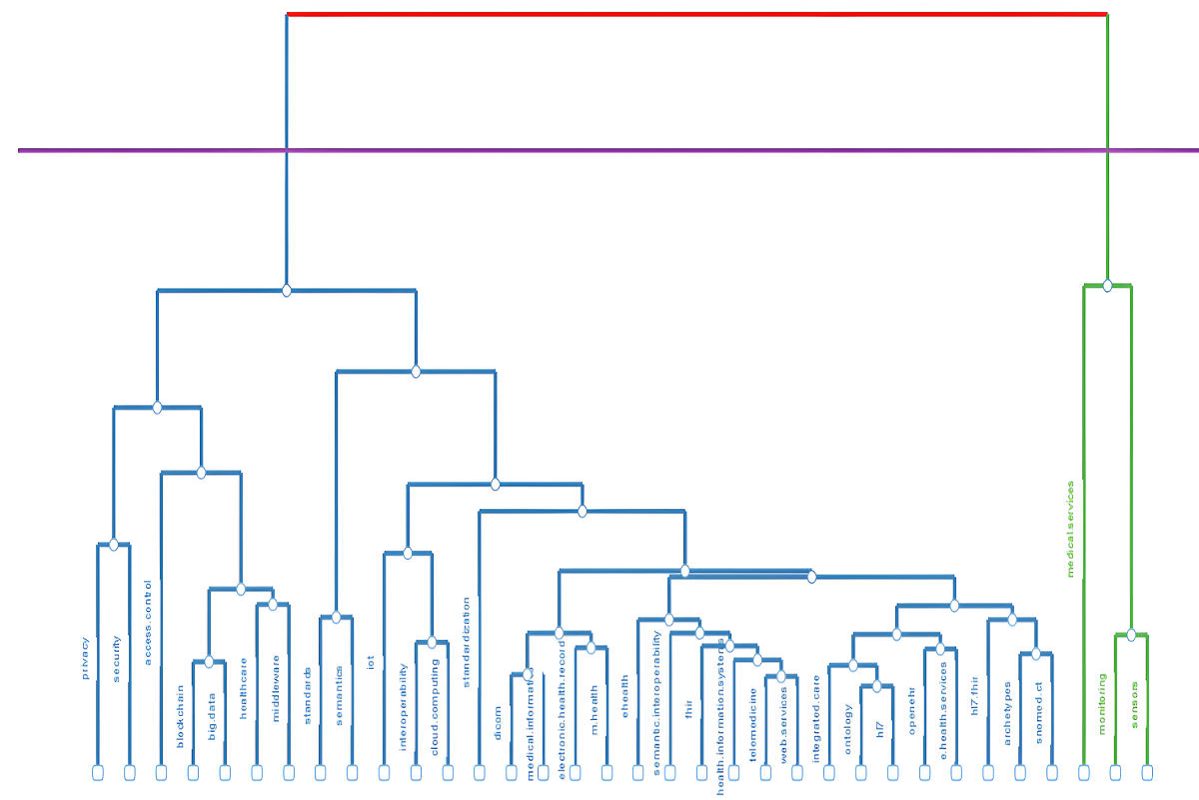
Hierarchy of topics of interoperability in health care. FHIR: Fast Healthcare Interoperability Resources; HL7: Health Level Seven; IoT: Internet of Things.

In this section, we analyze the conceptual classification and structure of this domain using factor analytic techniques, specifically multiple correspondence analysis. This evaluation yielded a 2-factor solution, encompassing 2 dimensions that account for 59.46% of the total variance (inertia). The first dimension contributed 36.78% to the inertia, while the second contributed 22.68% (see Figure 5). This 2-factor solution was corroborated by the topic dendrogram (see Figure 6). Most themes were associated with the first factor. The second factor encompassed only 3 elements: sensors, monitoring, and medical services.

**Figure 7:**
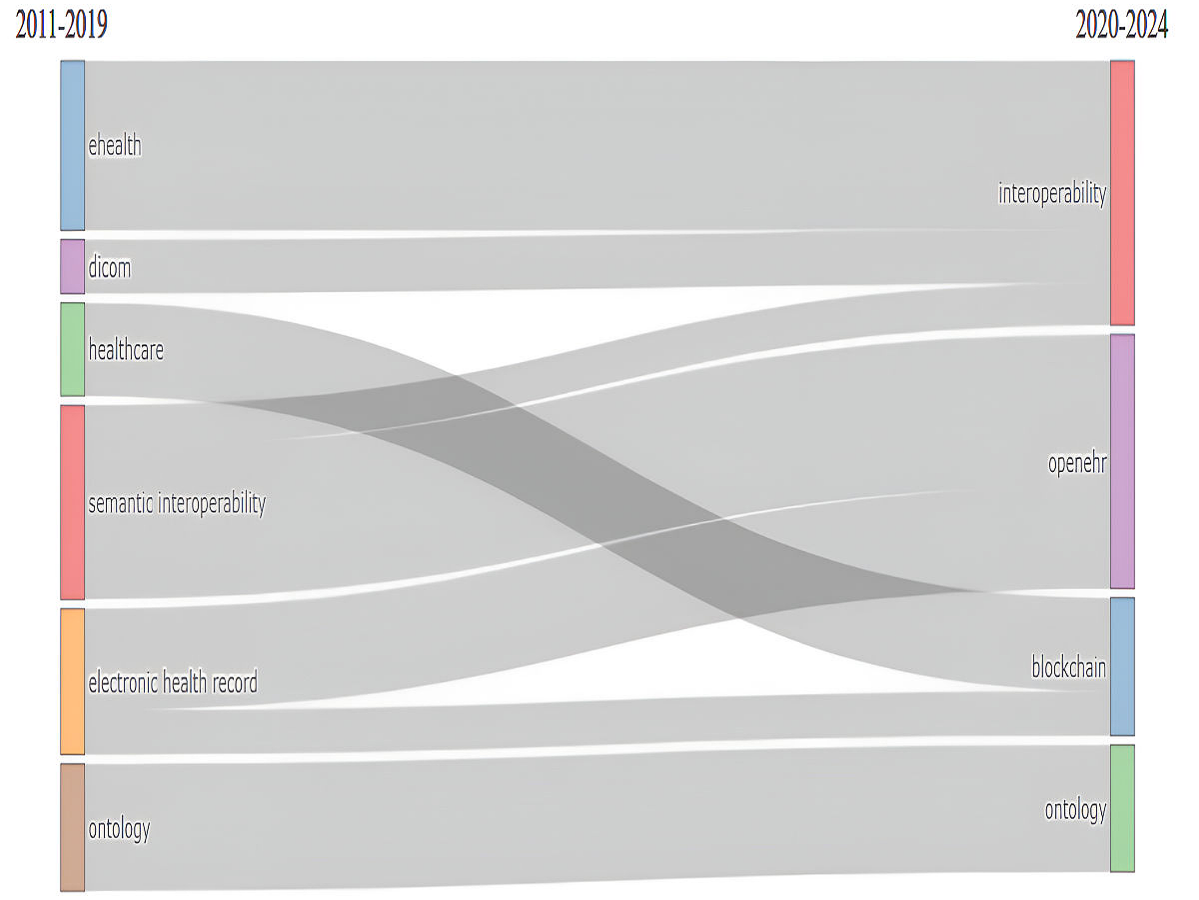
Thematic evolution of interoperability in health care.

The thematic evolution of this field, with a critical turning point in the year 2020, is presented in Figure 7. In the initial phase, 6 themes characterized the domain: eHealth, digital imaging and communication medicine (DICOM), health care, semantic interoperability, EHRs, and ontology. By 2020, ontology persisted as a key term, while the other themes were replaced by interoperability, openEHR, and blockchain.

### Literature Review

The literature review followed a systematic process [35,36] that encompassed 3 iterations. The initial iteration focused on documents with contributions of 1.0 or higher, resulting in a total of 28 articles, including both journal articles and gray literature. Notably, 15 out of 28 (54%) samples consisted of conference articles, indicating that the aim of achieving a balanced and representative sample—with journal articles as the majority—was not fulfilled. Therefore, additional iterations were deemed necessary to ensure category saturation. As a result, it was decided to incorporate 2 more iterations.

The second iteration included only journal articles, selected based on their contribution to the factorial solution, adding 35 journal articles to the sample. The third iteration comprised a final selection of 23 conference articles, primarily chosen for their contribution to the factorial solution and their availability. Unfortunately, we encountered access issues with 7 articles—1 from the first iteration, 2 from the second, and 4 from the third.

**Figure 8:**
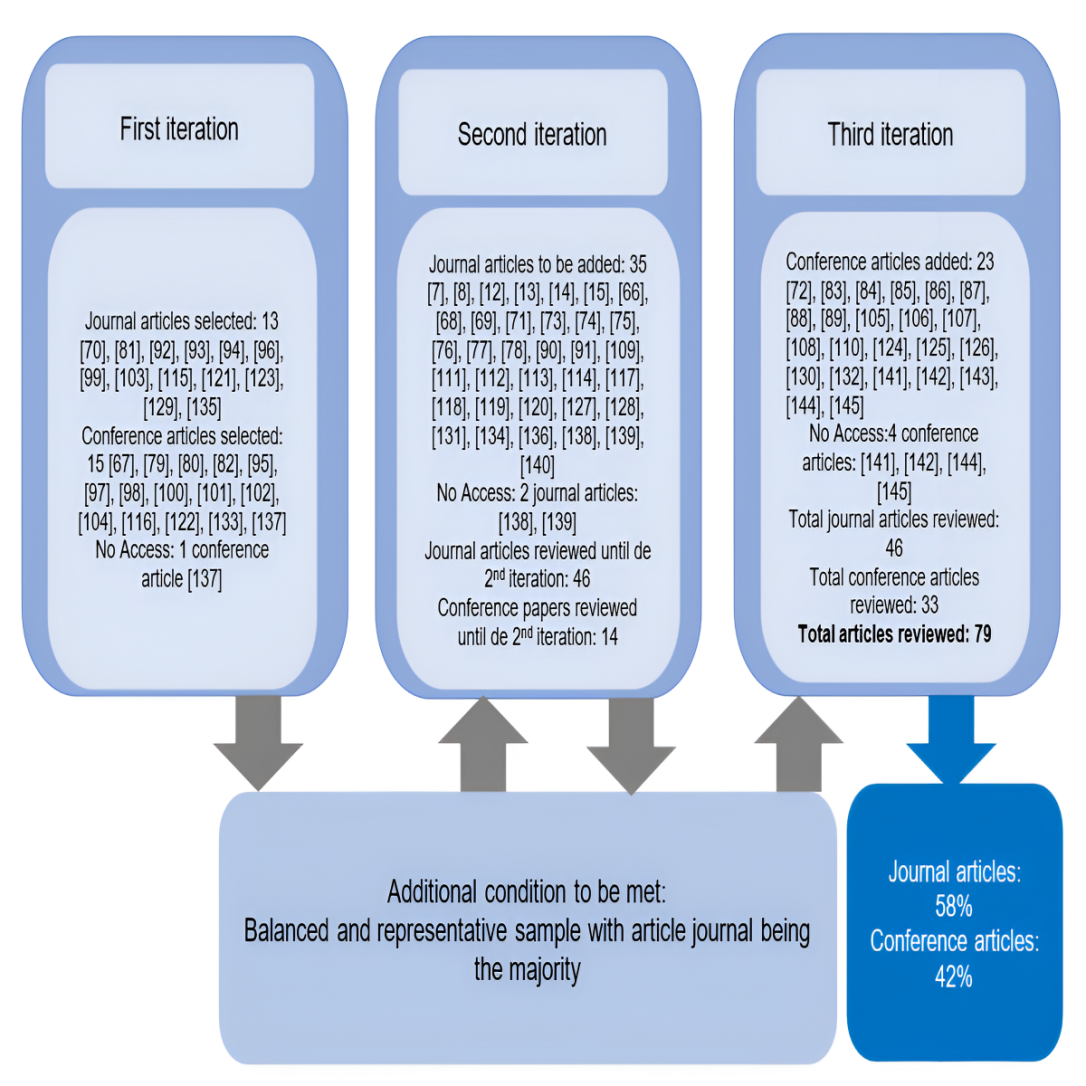
Systematic process for facet taxonomy development.

Ultimately, the review included a total sample of 79 out of the 370 (21.4) articles. Among these, 46 of the 79 (58%) documents were journal articles, while 33 of the 79 (42%) were conference papers. The list of reviewed articles can be found in Multimedia Appendix 3, and Figure 8 (see also [7,8,12-15,66-145]) illustrates the outlined systematic procedure.

**Figure 9:**
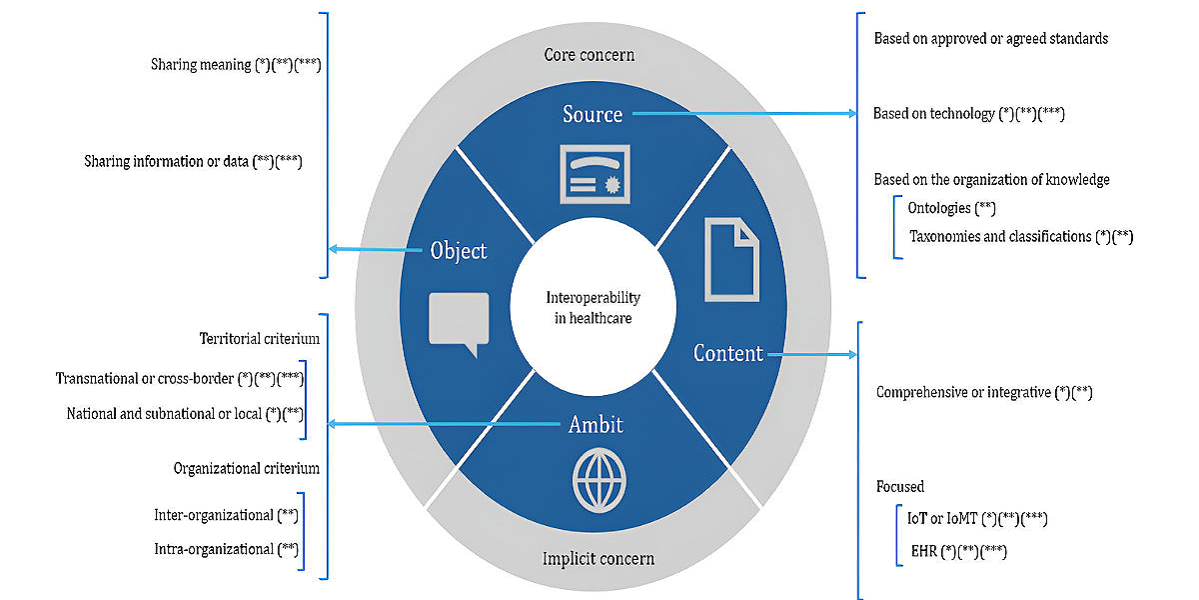
Higher order facets of interoperability in health care. EHR: electronic health record; IoMT: Internet of Medical Things; IoT: Internet of Things.

Following our analysis, we confirmed that saturation was achieved. All facets were identified within the first and second iterations, with no new facets introduced exclusively in the third iteration (see Figure 9). Additionally, all other established conditions were met. Therefore, we concluded that the objective was fulfilled after completing the 3 iterations.

In developing the facets, we reviewed the themes identified in the network and cluster analyses. Three facets—Content, Object, and Source—represented these themes and were designated as core concerns. The content involved the technology primarily intervened (encompassing, for example, the themes EHR and IoT). Object referred to what is shared (reflecting themes such as semantic interoperability and semantics). Source pertained to the mechanisms used to achieve interoperability (involving themes such as blockchain and standards, among others). Consequently, these 3 facets were considered core concerns. However, an additional facet, named Ambit, emerged from the literature review and was regarded as an implicit concern. Figure 9 illustrates the 4 higher-order facets and their subfacets, extending to the third degree, and Textbox 3 summarizes the key findings.

**Summary of key findings on higher-order facets of interoperability in the health care taxonomy.**

**1. Facet 1. Object**

- Sharing meaning (facet 1.1) [12,71-76]
- Sharing data or information (facet 1.2) [14,72,77,78]

**2. Facet 2. Source**

- Based on approved or agreed standard (facet 2.1) [13,74,79-95]
- Based on technology (facet 2.2) [75,79,80,86,94-116]
- Based on the organization of the knowledge—not on an agreed or approved standard (facet 2.3)
  - Ontologies (facet 2.3.1) [14,71,73,117-119]
  - Taxonomies or classifications (facet 2.3.2) [73,75]

**3. Facet 3. Content**

- Comprehensive or integrative (facet 3.1) [83,100,103]
- Focused (facet 3.2)
  - Electronic health record (facet 3.2.1) [8,74,77-81,96,97,107,118,120-122]
  - Internet of Things or Internet of Medical Things (facet 3.2.2) [7,72,75,76,79,82-84,86,95,98,99,103,105,107,115,116,123-131]

**4. Facet 4. Ambit**

- Territorial criterion (facet 4.1) [15,109]
  - Transnational or cross-border (facet 4.1.1) [7,12,15,74,77,110,130,132]
  - National and subnational or local (facet 4.1.2) [13,87,102,109,120,122,133-136]
- Organizational criteria (facet 4.2) [77]
  - Interorganizational (facet 4.2.1) [77,96]
  - Intraorganizational (facet 4.2.2) [97]

### Higher-Order Facets of Interoperability in Health Care Taxonomy

#### Facet 1. Object

##### Overview

This facet alludes to what is shared. In this regard, we adopted the concept of interoperability as the extent to which devices and systems exchange data [113] and meaningful information among them [11,113], allowing systems to work together [113]. Thus, we follow the perspective of system-to-system interoperability [14].

It is appropriate to note that the literature identifies various elements that may be shared. At times, the distinction between what is exchanged and who performs this activity is not sufficiently clear or consistent. This ambiguity has led to classifications that include organizations [99] and individuals [14]. Some authors adopt a broader concept of interoperability that encompasses social systems [131] and legal interoperability [15,73]. While we acknowledge the importance of these perspectives, they were not fully represented in our review. Therefore, we did not include them in the taxonomy.

##### Sharing Meaning (Facet 1.1)

Sharing meaning is achieved through semantic interoperability [12,66-70,146]. It aims to ensure the unambiguous understanding and interpretation of data by machines [71]. This level of understanding may range from partial to full semantic comprehension [113]. Controlled vocabularies [68], including terminologies, classifications, and ontologies [66,68,111], are central to this concept. Standards such as OpenEHR and HL7 v3 are proposed to facilitate interoperability. These standards emphasize the importance of a common model and the need for flexibility. As a result, standards such as OpenEHR use common reference models and archetypes to describe medical knowledge [112]. The common reference model consists of a predefined set of classes that form the structure of an EHR within the OpenEHR framework [112], while archetypes incorporate specific controlled-formal vocabularies—known as domain concepts—into the model [112]. These domain concepts are created in consultation with domain experts (eg, physicians) and can be either newly developed or reused [112]. For example, in the proposal for a cardiac surgery system based on OpenEHR, the authors reused existing archetypes such as “Patient admission and clinical synopsis” and developed new ones such as “Cardiac surgery” and “Angioplasty cardiac,” in addition to using the classes from the common reference model. Despite these promising features, the authors acknowledge limitations in the standards and propose alternatives, such as multilevel modeling [68].

##### Sharing Data or Information (Facet 1.2)

Sharing data or information encompasses both the exchange of messages between systems [14]—which does not necessarily involve understanding their meanings [14]—and the transmission techniques used [71]. The transmitted data are technically readable by the recipient [7]. Some authors distinguish between data sharing (technical interoperability) and information sharing (syntactic interoperability) [72]. The present subfacet encompasses both. Examples of protocols for data sharing include JSON [67] and XML-based protocols [67].

#### Facet 2. Source

##### Overview

This facet refers to the mechanisms used to achieve interoperability. Although standardization may appear to be a driver for interoperable solutions [13], the literature acknowledges its limitations and notes that it does not guarantee interoperability [70,76,113]. Therefore, this facet encompasses standards along with other sources that contribute to achieving interoperability.

##### Based on Approved or Agreed Standard (Facet 2.1)

Standards are defined sets of shared vocabulary or expectations among systems or devices [73]. The literature acknowledges the relevance of standards in achieving interoperability but also highlights their limitations. For instance, some authors argue that the existence of competing or incompatible standards can increase the complexity of the topic [13,69,99]. The coexistence of multiple protocols and standards—and their various versions, such as HL7 v2 and HL7 v3 [132]—to be applied within the same solution, combined with the need for efficient and resource-saving implementations, has led to proposals aimed at harmonizing or adapting them [75,77,78,84,86,87,95,118,123,132]. Then, the authors elaborated solutions that combine these standards [84,88,123], and proposed methods for selecting the most suitable combination to enhance interoperability rather than diminish it [13], as well as for adapting them to local contexts [112].

Following the literature [73], health care–focused standards can be categorized into vocabulary or terminology, for example, SNOMED-CT (Systematic Nomenclature of Medicine—Clinical Terms), LOINC (Logical Observation Identifiers Names and Codes), UCUM (Unified Code for Units of Measure), International Classification of Diseases (ICD) [71]; content (eg, HL7); information transport (eg, DICOM [75]); clinical document architecture (eg, CDA, one of HL7s standards); and structure tools (eg, FHIR, OpenEHR) [73]. A simpler classification organizes them into data and device standards [133]. Furthermore, some standards are more suitable for specific diseases. For example, DICOM—when complemented with other standards—holds particular relevance in ophthalmology, where imaging is central to diagnosis and treatment [75]. Other diseases discussed in various studies include dementia [131], infections [71], tuberculosis [68], and epilepsy [101], among others.

Some standards are widely recognized at the international level [133], with OpenEHR and FHIR being notable examples. Additionally, the literature identifies HL7, IHE (Integrating the Healthcare Enterprise), CEN (European Committee for Standardization [Comité Européen de Normalisation]), ISO (International Organization for Standardization), OpenEHR, IHTSDO (The International Health Terminology Standards Development Organization), and DICOM as the major international organizations that aim to establish standards for interoperability in health care [113,133]. By contrast, some standards or strategies emerge within the context of a specific country. For instance, an Italian national-level data-sharing proposal facilitates the interoperability of general practitioners’ EHRs with a central domain [81,116]. This proposal applied 16 extractors—or termed data miners—that cover a high percentage of general practitioners’ software to enable interoperability at the national level [81]. Its effectiveness was evaluated from both technical and user perspectives, yielding satisfactory results [81]. The literature also highlights the challenges involved in harmonizing national, regional, and transnational standards [130].

It is also relevant to mention that certain design principles or architectural styles, although not officially approved as standards, are referenced and adopted in the literature as tools to support interoperability. One example is the representational state transfer architectural style [82,83]. For instance, representational state transfer was used to develop a solution for interoperable IoT systems based on web technologies [82]. Finally, additional articles that referred to this facet are [74,76,79,80,85,89].

##### Based on Technology (Facet 2.2)

Blockchain, middleware, cloud computing, and gateways are technologies commonly used to achieve or enhance interoperability. They can be used in combination with standards but are not necessarily dependent on them. Some proposals even support the concept of open, nonstandard-based interoperability [99], incorporating various elements to provide interoperable solutions [99].

Blockchain is a distributed ledger that enhances patients’ data ownership [73,100], as well as privacy and security [97], through its cryptographic features. Simultaneously, the literature has highlighted its role in enabling interoperability [73,74,90,91,93,94,96,100,105,120]. In this context, the use of interplanetary file systems as an off-chain storage solution [90], encryption techniques such as fully homomorphic encryption [90], and smart contracts [74,91,105] have also been proposed. For example, 1 system employs remote data storage as a private blockchain application, incorporating the most prominent blockchain platforms—Ethereum and Hyperledger Fabric [73]. This proposal enabled the adoption of blockchain and the integration of legacy data from physicians and health care institutions [73]. Its proof of concept was applied to medical clinic data from Bucharest and yielded satisfactory results [73].

Middleware is also recognized as a technological solution for achieving interoperability [82,101,109,110,117,131,134,135]. In the context of IoT sensors, middleware facilitates data retrieval from devices that use diverse proprietary protocols [117]. Additionally, middleware offers desirable levels of adaptability, flexibility, and efficiency in resource usage [109]. Despite its advantages for interoperability, the literature highlights concerns regarding the proliferation of middleware platforms lacking common protocols [130].

Cloud computing [12,76,81,95,106-109,116], along with its predecessor, grid computing [134], and its decentralized variants, such as fog computing [120] and mobile edge computing [95], contribute to interoperability through their protocols [12,76] and technical characteristics—such as offering a single point of access [108]. These technologies facilitate integration across systems and devices, enhancing interoperability in distributed environments. However, the literature also notes challenges associated with cloud-based solutions, including architectural heterogeneity in complex environments such as federated clouds [98] and limitations related to cloud storage capacity and reliability [102]. These challenges may lead to a lack of interoperability between cloud environments [80,98]. To address intercloud interoperability issues, the literature proposes solutions such as brokering services based on web standards [80,107], as well as the application of blockchain technologies [102].

Gateways [70,88,89,92,104,125] can also translate protocols, filter data, and process it to provide interoperability [92]. Similarly, plug-and-play solutions that function as gateways are also sources of interoperability [70]. Some authors propose portable central processing hubs that identify data from different devices without requiring ontologies or physical changes in systems [121]. Others base their interoperable solutions on the data warehouse [69]. Finally, additional articles that referred to this facet are [80,95,103].

##### Source Based on the Organization of the Knowledge—Not on an Agreed or Approved Standard (Facet 2.3)

The literature acknowledges controlled vocabularies as a relevant tool for achieving interoperability [68], particularly semantic interoperability. Classifications, taxonomies, and ontologies are types of controlled vocabularies [68]. Some controlled vocabularies may be novel and created for specific purposes, while others are required to adopt a standard—such as OpenEHR—for particular diseases or local contexts [68,84]. For this reason, different selection criteria, techniques, and development approaches are applied to enhance their performance [68]. The subfacets for this source are described in Textbox 4.

**Source based on the organization of the knowledge—not on an agreed or approved standard.**

1. Ontologies (subfacet 2.3.1)
  Ontologies provide semantic interoperability [66] by offering concepts and the relationships among them [66], thereby supporting a rational argument [68]. Unlike technical solutions designed to respond to protocols developed by a set of providers, the aim in this case is to embody a specific knowledge domain [14,66] or to adapt it [8,84,112].
  Different approaches can be used to develop ontologies, such as applying acknowledged methodologies [66,111] and languages [67,77]. Some proposals reuse existing ontologies [111] to improve the ontology-building process [66]. Nevertheless, certain ontologies are primarily novel developments [14,67]. Other studies have also incorporated archetypes into the standard to adapt it to specific requirements [8]. The translation of existing archetypes into other languages has also been considered [77]. Protégé and Pellet Reasoner are implementation tools used to test the proposed ontologies [67].
  The literature also acknowledges vagueness—using imprecise concepts to describe ideas—and uncertainty as inherent features of the medical domain [113]. This has led to the development of fuzzy ontologies [113]. Among other reasons, the authors argue that fuzzy semantics may be particularly suitable for this domain, as it aligns more closely with human thinking and reasoning, can handle vague and unclear data, and is capable of managing both structured and unstructured data [113].
2. Taxonomies or Classifications (subfacet 2.3.2)
  Taxonomies or classifications use terminologies in an aggregated manner based on a certain level of abstraction [68]. They may include both classified and hierarchical concepts. Applying taxonomies in specific contexts may require additional effort. For instance, implementing the International Classification of Diseases, 10th Revision (ICD-10) term subset in the Brazilian context involved careful selection and technical development using multilevel health information modeling [68].
  Fuzzy logic–based classifications have also been recommended [70]. For example, fuzzy logic can compare vital parameters extracted from different wearable devices with an established keyword list [70]. This classification approach achieved a high level of accuracy [70]; however, it was advisable to complement it with natural language learning techniques [70].

#### Facet 3. Content

##### Overview

This facet refers to the HIT that is primarily targeted. Most authors focused on improving the interoperability of a specific HIT—even if they mentioned others—while some aimed to integrate multiple HITs [77,94,97]. We refer to the former as focused and the latter as comprehensive or integrative. Additionally, we identified some focused solutions with a specific goal in mind, such as integrating legacy systems or data [93,96].

##### Comprehensive or Integrative (Facet 3.1)

The compatibilization of specific HITs has also been proposed. The literature highlights the challenges in achieving interoperability between EHR and IoT systems [77,94]. These challenges stem from the complexity and volume of the data, the existence of multiple proprietary protocols, and the topic-focused development of standards such as OpenEHR (for EHR) and HL7 (for IoT), which require data translation to ensure compatibility between them [77]. Additional issues relate to security and privacy concerns [94]. One proposed solution involved extending existing standards for each HIT to enable their joint use [77]. Furthermore, the use of middleware has also been suggested to improve communication between IoT and EHR systems [94,97], as sensors generate data that are stored in and retrieved from EHRs [94], while also enhancing security.

##### Focused (Facet 3.2)

The most frequently cited HITs are IoT (or Internet of Medical Things [IoMT]) and EHR. We based the classification of this subfacet on these 2 technologies. Telemedicine [126], which involves the use of HIT to provide health care services remotely [12], commonly incorporates IoT [122] or IoMT technologies [12]. For this reason, we did not consider telemedicine an independent subfacet. Textbox 5 presents the subfacets of focused.

**Subfacets of focused.**

1. Electronic health record (subfacet 3.2.1)
  The literature distinguishes between personal health records [73,90,101] and electronic health records (EHRs) [73,90]. While we acknowledge this distinction, for the purposes of this study, the 2 terms will be used synonymously under the umbrella of EHR. A central concern in this area involves data ownership [73], data security and privacy [74], and compliance with regulations. OpenEHR standards have been widely adopted to support interoperability [71,76]. Interoperability issues can arise from differences in software and programming languages [71], the comprehensiveness of the software, the type of data involved (eg, structured vs unstructured) [71], and other related factors. It is also important to highlight the relationship between EHRs and emerging developments such as digital twins [72]. The literature notes that EHRs can be connected to, enhanced by, or even replaced with digital twins [72].
  Integrating existing data from EHR legacy systems has also been identified as a significant challenge [71]. For instance, various frameworks and solutions have been proposed to address data migration from centralized systems to decentralized, blockchain-based architectures [73,115]. Other studies have tackled the issue of maintaining and reusing information originally created in traditional SQL-based systems when transitioning to new systems using openEHR, which are oriented toward NoSQL document structures [8]. OpenEHR storage control has also been used to ensure compatibility with legacy systems [77]. Finally, additional articles that referred to this facet are [69,75,91,112,114,116].
2. Internet of Things or Internet of Medical Things (subfacet 3.2.2)
  Internet of Things (IoT) or Internet of Medical Things (IoMT) refers to a network of devices equipped with sensors [12] that can collect health information from individuals [117]. Interoperability within IoT or IoMT presents specific challenges, such as the use of various proprietary protocols across sensors and devices [12,67,109,117,119], and data heterogeneity [67], which necessitates data cleaning before sharing to prevent measurement errors [12]. Additionally, because sensors have limited resources, any proposed solution must be resource efficient [70,92,134]. Plug-and-play [70] and portable [121] solutions have also addressed these efficiency challenges. Authors further propose both direct and indirect integration of devices: the former is implemented directly on the device, while the latter uses connectors that define communication protocols [89].
  Wearable devices play a central role [12]. This includes devices used for remote health care services [80] as well as those employed within health care facilities, including critical settings such as emergency care [122]. These solutions can be used to monitor activities performed by healthy individuals, such as physical activity [99], but they can also be applied to specific conditions, including diabetes [146] and eating disorders [99].
  The literature also raises concerns about preserving existing data in the context of IoT or IoMT systems [93,96]. Blockchain-based solutions have proposed the progressive migration of these data [93,96]. However, this migration presents several challenges, including architectural differences, data types, user types, and transaction synchronization, among others [93,96]. Finally, additional articles that referred to this facet are [7,70,73,76-78,97,99,101,110,118,120,123-125].

#### Facet 4. Ambit

##### Overview

This facet refers to the scope addressed by interoperability projects. On the one hand, authors referred to a territorial perspective [15,103]; on the other hand, the literature emphasized the role of organizations [103]. We also propose that this classification offers deeper insights into the concepts of transverse interoperability—collaboration among stakeholders involved with the same patient—and vertical interoperability—the integration of databases and national health systems [81].

##### Territorial Criterion (Facet 4.1)

The territorial criterion refers to physical space. The literature classifies this criterion in various ways. Some authors divide it into supra-national, national, regional, and local levels [103], while others distinguish primarily between supra-national, national, and subnational levels [15]. In this study, we follow the latter classification. The subfacets are described in Textbox 6.

**Subfacets of territorial criterion.**

1. Transnational or cross-border (subfacet 4.1.1)
  In this subfacet, the solutions highlight the importance of connecting to the international landscape [7,12,104]. One example is the PRAISE network, which brought together experts from multiple countries aiming to evaluate health-associated infections [71]. Specific interoperability challenges arise in the transnational context—for instance, differing legal environments [69,124], and the need for various local adaptations [71]. Within the European Union, interoperability encounters additional difficulties, such as variations in national regulations and their stringency [124,126], differing levels of implementation and interpretation of European directives [15,124,126], and the requirement to maintain national infrastructures [104].
2. National and subnational or local (subfacet 4.1.2)
  Some studies are country-specific and may aim to assess interoperability across an entire national territory [81,114,116,127-129] or a part of it [103,136]. These inquiries may be driven by national initiatives or strategies formulated by governmental agencies [114,127], addressing specific issues such as older adult health care [127] or challenges related to national-level regulations [116]. In some cases, authors have identified national-level eHealth systems [96] and their interoperability as a central concern. In other analyses, authors assess the situation and propose frameworks or guidelines [13,114,127]. A subnational example is the development of a neurosurgical telecounseling network in Veneto, an Italian region [136]. In this case, national legislation on privacy and security had to be adequate, and the solution’s architecture reflected the region’s organizational structure. It was divided into 7 groups—1 for each province—while also incorporating and adapting international standards where necessary [136].
  These proposals must address not only the technical aspects of the solution but also take into account the specific realities and limitations of the country where the solution will be implemented [13,103,114,127,129]. Countries mentioned in the literature include Jordan [114], the United Kingdom [127], Brazil [68], Kenya and other African nations [13], Pakistan [120], Tanzania [129], and Italy [81]. The authors also emphasized the challenges that developing countries, such as Tanzania, face in building interoperable systems [129]. Finally, additional articles that referred to this facet are [96,130].

##### Organizational Criteria (Facet 4.2)

We considered organizational and territorial levels as distinct subfacets, as organizations can operate at subnational, national, or international levels and establish connections with other organizations [71]. Textbox 7 describes these subfacets.

**Subfacets of organizational criteria.**

1. Interorganizational (subfacet 4.2.1)
  Solutions may involve various health care institutions, such as different hospitals [71,90], or other stakeholders within the health care supply chain. These proposals are not limited by territorial boundaries but aim to connect different organizations. The degree of coordination can vary and may include centralized solutions [71] or decentralized ones, such as those based on blockchain [90].
2. Intraorganizational (subfacet 4.2.2)
  Some studies focus not primarily on information sharing among organizations but rather on interoperability between different components of a health information system [91], such as electronic health records, clinical medical information, and clinical decision support systems [91].

## Discussion

### Principal Findings

The intricate landscape of knowledge in the field of comprehensive health care interoperability is clearly illustrated by 2 distinct clusters of terms that frequently converge. The results first highlight the importance of eHealth and interoperability in shaping the field. This finding aligns with previous studies on the concept of eHealth, which emphasize understanding “health” as an ongoing process rather than a fixed outcome, and stress the role of technology as a tool to support—rather than replace—humans [147]. The 2 clusters also reflected the focus on 2 HITs associated with interoperability concerns: (1) EHRs and (2) IoT. Additionally, standards development—as a response to interoperability limitations, including those related to semantic interoperability—was grouped within the first cluster, along with EHR. This highlights the challenges surrounding EHR interoperability and underscores the importance of semantic interoperability and standard-based solutions for this HIT. By contrast, emerging technologies—such as blockchain, middleware, and cloud computing, which can also contribute to interoperability—along with privacy and security, formed the second cluster, centered around IoT. This grouping reflects the complexity of interoperability in the IoT domain, which encompasses concerns related to privacy and security, as well as a strong interest in leveraging cutting-edge technologies to address these challenges.

Factorial analysis has revealed 2 fundamental factors that shape this dynamic field. Central to these are key concepts such as EHRs, FHIR, and semantic interoperability, which collectively form the backbone of innovation and collaboration in health care data exchange. Notably, the factorial solution aligned with the cluster analysis in identifying 2 distinct conceptual groupings that characterize the field. However, the specific membership of elements within each group differed. This discrepancy can be attributed to the fact that cluster and factor analysis partition variance through different methodological approaches, potentially leading to variation in how elements are grouped.

In addition, the thematic arrangement observed in the post–COVID-19 context was expected, as health care systems were compelled to prioritize interoperability to enable pervasive and responsive services [148]. The urgency to contain the pandemic underscored the critical need to manage a wide range of population health data generated by diverse technologies and systems [148]. Consequently, interoperability emerged as a central concern. Within this framework, EHRs gained heightened significance due to their role in systematically capturing individual health information. Notably, openEHR—a standard that facilitates multiple levels of interoperability, including semantic interoperability, and integrates domain knowledge from medical experts—maintained and even strengthened its relevance during this period [149]. The literature also underscored the recognized importance of the IoT during the COVID-19 pandemic. However, the widespread implementation of IoT solutions raised significant concerns related to security and privacy. In this context, blockchain emerged as a promising technology, offering features such as decentralization, traceability, transparency, and trustworthiness to address these challenges effectively [150]. Despite the considerable disruptions caused by the pandemic, the concept of ontology retained its relevance, continuing to attract scholarly attention and exploration.

The highest-order facets of this multifaceted domain—namely, object, source, content, and ambit—form a foundational framework that weaves a comprehensive understanding essential for advancing health care interoperability and ultimately enhancing patient care.

### Limitations and Further Studies

We adopted a systematic approach to data selection and assessment to minimize bias in our research. Nonetheless, several limitations must be acknowledged. First, in our effort to achieve a comprehensive analysis, we consulted and integrated data from multiple databases. Despite our best efforts to make well-informed decisions regarding data merging, cleaning, and preparation, these processes inherently involved data wrangling and manipulation, which may have introduced some bias. Second, our study is temporally constrained, as it focuses on articles published from 2011 onward. While this scope enabled us to capture the most recent and relevant developments, it also limited the inclusion of earlier foundational work. Third, although our literature review was conducted systematically and informed by the outcomes of factor assessments, we recognize that the risk of selection bias may still be present. Fourth, we developed a taxonomy through a systematic literature review process, making our best efforts to avoid bias; however, this process was grounded in category saturation based on a sample. Thus, this taxonomy has yet to undergo evaluation. Conducting such an evaluation is crucial for its final application and, potentially, for propounding policies to improve levels of interoperability in health care. Additionally, there is a significant opportunity for future research to integrate the higher-order dimensions we have identified with existing specialized frameworks. By leveraging faceted taxonomies, we can provide richer, more nuanced descriptions of this complex topic, enabling the development of terminology that facilitates effective integration. Fifth, the creation of ontologies that enrich the semantic context of these identified facets holds great promise for advancing our understanding and application of interoperability in health care. Sixth, technologies used to provide interoperability from the perspective of the supply chain, such as blockchains [151-154], present limitations and barriers that require further study—in terms of both their capability to provide interoperability and their own interoperability. Additional studies are also suggested regarding regulation, which could be seen as a barrier to the standardization of interoperability [155].

### Comparison With Prior Work

A previously conducted bibliometric analysis on interoperability in health care [31] identified 3 clusters based on abstracts: eHealth information stakeholder needs—which included terms such as “electronic health record” and “standard”—eHealth information quality assessment, and eHealth information technological governance trends—which incorporated terms such as “blockchain,” “privacy,” and “security.” By contrast, our study identified 2 clusters, with the term IoT acquiring high relevance. One potential reason for this discrepancy may stem from the selection criteria and the volume of data used—the earlier analysis assessed 63 articles with the highest citation counts [31]. Nonetheless, we concur that one of the clusters identified in our research pertains to EHR and standards, and the other to the use of advanced technologies, such as blockchain, to provide solutions, as well as to concerns such as privacy and security.

In alignment with the survey on interoperability requirements [9], our study acknowledges the significance of standards as a crucial attribute of the topic. The classification of standards presented in the aforementioned survey could enrich the subfacet related to the thematic scope of standards.

Ultimately, we believe that the contributions of our study—specifically, the classification of topics through cluster and network analyses, along with the classification of attributes based on a comprehensive review of selected studies—can offer valuable insights for reevaluating the ongoing debate on the classifications and levels of interoperability in health care.

### Conclusion

Our research compellingly illustrates the vast domain of interoperability in health care, analyzed through the lens of supply chain management. eHealth emerged as a pivotal topic within this domain of knowledge, and the interoperability of EHR and IoT represents 2 key thematic categories encompassing several efforts. Finally, 4 critical attributes of interoperability were identified: (1) source, (2) content, (3) ambit, and (4) object.

## Appendix

Multimedia Appendix 1 PRISMA-S (Preferred Reporting Items for Systematic reviews and Meta-Analyses—Search extension) checklist.

Multimedia Appendix 2 Quantitative analysis (n=370 articles).

Multimedia Appendix 3 Qualitative analysis (complete list).

## Abbreviations

CEN: European Committee for Standardization (Comité Européen de Normalisation)
DICOM: digital imaging and communication medicine
DOI: digital object identifier
EHR: electronic health record
FHIR: Fast Healthcare Interoperability Resources
HIT: health information technology
HL7: Health Level Seven
ICD-10: International Classification of Diseases, 10th Revision
IHE: Integrating the Healthcare Enterprise
IHTSDO: The International Health Terminology Standards Development Organization
IoMT: Internet of Medical Things
IoT: Internet of Things
ISO: International Organization for Standardization
LOINC: Logical Observation Identifiers Names and Codes
SNOMED-CT: Systematic Nomenclature of Medicine—Clinical Terms
UCUM: Unified Code for Units of Measure
WoS: Web of Science

## References

[1] Amin D, Vandenbroucke P (2023). Advancing patient-centricity in Medical Affairs: A survey of patients and patient organizations. Drug Discovery Today.

[2] Srivastava, A. P., Sharma G., Shrivastava A., Singh Y., Salim Mohd. (2023). Bioinspired Sensory Gating in the Artificial Vision System.

[3] Ashkenazy R (2020). Building the case for developing a medical affairs patient-centric framework collaboratively. Drug Discovery Today.

[4] Toni M, Mattia G, Pratesi CA (2024). What’s next in the healthcare system? The contribution of digital innovation in achieving patient-centricity. Futures.

[5] Srivastava S, Singh RK (2020). Exploring integrated supply chain performance in healthcare: a service provider perspective. BIJ.

[6] Cardoso DMJL, de SWL, Pires LF, do PAF (2016). A methodology based on openEHR archetypes and software agents for developing e-health applications reusing legacy systems. Comput Methods Programs Biomed.

[7] Garai I, Péntek I, Adamkó A, Németh A (2017). Methodology for clinical integration of e-Health sensor-based smart device technology with cloud architecture. Pollack Periodica.

[8] Khennou F, Chaoui NEH, Khamlichi YI (2019). A migration methodology from legacy to new electronic health record based OpenEHR. International Journal of E-Health and Medical Communications.

[9] Torab-Miandoab A, Samad-Soltani T, Jodati A, Rezaei-Hachesu P (2023). Interoperability of heterogeneous health information systems: a systematic literature review. BMC Med Inform Decis Mak.

[10] Rezaei R, Chiew TK, Lee SP, Shams Aliee Z (2014). Interoperability evaluation models: A systematic review. Computers in Industry.

[11] Hazra A, Adhikari M, Amgoth T, Srirama SN (2021). A Comprehensive Survey on Interoperability for IIoT: Taxonomy, Standards, and Future Directions. ACM Comput. Surv.

[12] Garai Á., Péntek I., Adamkó A. (2019). Revolutionizing Healthcare with IoT and Cognitive, Cloud-based Telemedicine. APH.

[13] Adebesin F, Kotzé P (2017). A process for developing an e-health standards selection method artefact using design science research. JDR.

[14] Mouttham A, Kuziemsky C, Langayan D, Peyton L, Pereira J (2011). Interoperable support for collaborative, mobile, and accessible health care. Inf Syst Front.

[15] Kautsch M, Lichoń M, Matuszak N (2016). Setting the scene for the future: implications of key legal regulations for the development of e‐health interoperability in the EU. Health Planning & Management.

[16] Palojoki S, Lehtonen L, Vuokko R (2024). Semantic Interoperability of Electronic Health Records: Systematic Review of Alternative Approaches for Enhancing Patient Information Availability. JMIR Med Inform.

[17] Carmagnola F, Cena F, Gena C (2011). User model interoperability: a survey. User Model User-Adap Inter.

[18] House A, Power N, Alison L (2013). A systematic review of the potential hurdles of interoperability to the emergency services in major incidents: recommendations for solutions and alternatives. Cogn Tech Work.

[19] Villarreal ERD, Garcia-Alonso J, Moguel E, Alegria JAH (2023). Blockchain for Healthcare Management Systems: A Survey on Interoperability and Security. IEEE Access.

[20] Sonkamble RG, Phansalkar SP, Potdar VM, Bongale AM (2021). Survey of Interoperability in Electronic Health Records Management and Proposed Blockchain Based Framework: MyBlockEHR. IEEE Access.

[21] de Mello BH, Rigo SJ, da Costa CA, da Rosa Righi R, Donida B, Bez MR, Schunke LC (2022). Semantic interoperability in health records standards: a systematic literature review. Health Technol (Berl).

[22] Moreno-Conde A, Moner D, Cruz WDD, Santos MR, Maldonado JA, Robles M, Kalra D (2015). Clinical information modeling processes for semantic interoperability of electronic health records: systematic review and inductive analysis. J Am Med Inform Assoc.

[23] Sasse J, Darms J, Fluck J (2022). Semantic Metadata Annotation Services in the Biomedical Domain—A Literature Review. Applied Sciences.

[24] Ait Abdelouahid R, Debauche O, Mahmoudi S, Marzak A (2023). Literature Review: Clinical Data Interoperability Models. Information.

[25] Ayaz M, Pasha MF, Alzahrani MY, Budiarto R, Stiawan D (2021). The Fast Health Interoperability Resources (FHIR) Standard: Systematic Literature Review of Implementations, Applications, Challenges and Opportunities. JMIR Med Inform.

[26] Nan J, Xu L (2023). Designing Interoperable Health Care Services Based on Fast Healthcare Interoperability Resources: Literature Review. JMIR Med Inform.

[27] Vorisek CN, Lehne M, Klopfenstein SAI, Mayer PJ, Bartschke A, Haese T, Thun S (2022). Fast Healthcare Interoperability Resources (FHIR) for Interoperability in Health Research: Systematic Review. JMIR Med Inform.

[28] Maritz R, Aronsky D, Prodinger B (2017). The International Classification of Functioning, Disability and Health (ICF) in Electronic Health Records. Appl Clin Inform.

[29] Haque AKMB, Arifuzzaman BM, Siddik SAN, Kalam A, Shahjahan TS, Saleena TS, Alam M, Islam MR, Ahmmed F, Hossain MJ (2022). Semantic Web in Healthcare: A Systematic Literature Review of Application, Research Gap, and Future Research Avenues. International Journal of Clinical Practice.

[30] Kinast B, Ulrich H, Bergh B, Schreiweis B (2023). Functional Requirements for Medical Data Integration into Knowledge Management Environments: Requirements Elicitation Approach Based on Systematic Literature Analysis. J Med Internet Res.

[31] Costa T, Borges-Tiago T, Martins F, Tiago F (2024). System interoperability and data linkage in the era of health information management: A bibliometric analysis. HIM J.

[32] Munn Z, Peters MDJ, Stern C, Tufanaru C, McArthur A, Aromataris E (2018). Systematic review or scoping review? Guidance for authors when choosing between a systematic or scoping review approach. BMC Med Res Methodol.

[33] Klarin A (2024). How to conduct a bibliometric content analysis: Guidelines and contributions of content co‐occurrence or co‐word literature reviews. Int J Consumer Studies.

[34] Caputo A, Kargina M (2021). A user-friendly method to merge Scopus and Web of Science data during bibliometric analysis. J Market Anal.

[35] Nickerson RC, Varshney U, Muntermann J (2012). A method for taxonomy development and its application in information systems. Eur J Inf Syst.

[36] Kundisch D, Muntermann J, Oberländer AM, Rau D, Röglinger M, Schoormann T, Szopinski D (2021). An Update for Taxonomy Designers. Bus Inf Syst Eng.

[37] Cheng C, Wang L, Xie H, Yan L (2023). Mapping digital innovation: A bibliometric analysis and systematic literature review. Technological Forecasting and Social Change.

[38] Imamoglu G, Topcu YI, Aydin N (2023). A Systematic Literature Review of the Blood Supply Chain through Bibliometric Analysis and Taxonomy. Systems.

[39] Rethlefsen ML, Kirtley S, Waffenschmidt S, Ayala AP, Moher D, Page MJ, Koffel JB (2021). PRISMA-S: an extension to the PRISMA Statement for Reporting Literature Searches in Systematic Reviews. Syst Rev.

[40] Page MJ, McKenzie JE, Bossuyt PM, Boutron I, Hoffmann TC, Mulrow CD, Shamseer L, Tetzlaff JM, Akl EA, Brennan SE, Chou R, Glanville J, Grimshaw JM, Hróbjartsson A, Lalu MM, Li T, Loder EW, Mayo-Wilson E, McDonald S, McGuinness LA, Stewart LA, Thomas J, Tricco AC, Welch VA, Whiting P, Moher D (2021). The PRISMA 2020 statement: An updated guideline for reporting systematic reviews. PLoS Med.

[41] Mills R, Mangone ER, Lesh N, Jayal G, Mohan D, Baraitser P (2024). Chatbots That Deliver Contraceptive Support: Systematic Review. J Med Internet Res.

[42] Ullah R, Asghar I, Griffiths MG (2022). An Integrated Methodology for Bibliometric Analysis: A Case Study of Internet of Things in Healthcare Applications. Sensors.

[43] Drongstrup D, Malik S, Aljohani NR, Alelyani S, Safder I, Hassan S (2020). Can social media usage of scientific literature predict journal indices of AJG, SNIP and JCR? An altmetric study of economics. Scientometrics.

[44] Lim WM, Kumar S, Donthu N (2024). How to combine and clean bibliometric data and use bibliometric tools synergistically: Guidelines using metaverse research. Journal of Business Research.

[45] Briones-Bitar J, Carrión-Mero P, Montalván-Burbano N, Morante-Carballo F (2020). Rockfall Research: A Bibliometric Analysis and Future Trends. Geosciences.

[46] Nikolić D, Ivanović D, Ivanović L (2024). An open-source tool for merging data from multiple citation databases. Scientometrics.

[47] Adeosun SO (2023). Trends in authorship characteristics and collaboration in pharmacy practice publications: 2011–2020. Research in Social and Administrative Pharmacy.

[48] Liman P, Anastasya K, Salma N, Yenny Y, Faradilla M (2022). Research Trends in Advanced Glycation End Products and Obesity: Bibliometric Analysis. Nutrients.

[49] Ullah F, Al-Turjman F (2021). A conceptual framework for blockchain smart contract adoption to manage real estate deals in smart cities. Neural Comput & Applic.

[50] Bender ME, Edwards S, von Philipsborn P, Steinbeis F, Keil T, Tinnemann P (2015). Using Co-authorship Networks to Map and Analyse Global Neglected Tropical Disease Research with an Affiliation to Germany. PLoS Negl Trop Dis.

[51] Merdietio Boedi R, Mânica S, Franco A (2023). Sixty years of research in dental age estimation: a bibliometric study. Egypt J Forensic Sci.

[52] Dreyer J, Bergmann JM, Köhler K, Hochgraeber I, Pinkert C, Roes M, Thyrian JR, Wiegelmann H, Holle B (2022). Differences and commonalities of home-based care arrangements for persons living with dementia in Germany – a theory-driven development of types using multiple correspondence analysis and hierarchical cluster analysis. BMC Geriatr.

[53] De Moor S, Vandeviver C, Vander Beken T (2020). Assessing the missing data problem in criminal network analysis using forensic DNA data. Social Networks.

[54] Hair Joseph F., Black William C., Babin Barry J., Anderson Rolph E. (2014). Multivariate Data Analysis. Pearson New International Edition 7th ed. Pearson.

[55] Chen C (2017). Science Mapping: A Systematic Review of the Literature. Journal of Data and Information Science.

[56] Chen C (2006). CiteSpace II: Detecting and visualizing emerging trends and transient patterns in scientific literature. J. Am. Soc. Inf. Sci.

[57] Tamasiga P, Mfuni H, Onyeaka H, Ouassou EH (2023). Green industrial policy as an enabler of the transition to sustainability: challenges, opportunities and policy implications for developing countries. Environ Dev Sustain.

[58] Brusco M, Steinley D, Watts AL (2024). Improving the Walktrap Algorithm Using -Means Clustering. Multivariate Behavioral Research.

[59] Brusco MJ, Steinley D, Watts AL (2022). On maximization of the modularity index in network psychometrics. Behav Res.

[60] Christensen AP (2024). Unidimensional community detection: A monte carlo simulation, grid search, and comparison. Psychological Methods.

[61] Bailey KD (1994). Typologies and Taxonomies. An Introduction to Classification Techniques. Sage Research Methods.

[62] Tzitzikas Y (2009). Faceted Taxonomy-Based Sources, in Dynamic Taxonomies Faceted Search. Springer Nature.

[63] Hennink MM, Kaiser BN, Marconi VC (2016). Code Saturation Versus Meaning Saturation. Qual Health Res.

[64] Bailón-Moreno R, Jurado-Alameda E, Ruiz-Baños R, Courtial JP (2005). The unified scientometric model. Fractality and transfractality. Scientometrics.

[65] Bailón-Moreno R, Jurado-Alameda E, Ruiz-Baños R, Courtial JP (2005). Bibliometric laws: Empirical flaws of fit. Scientometrics.

[66] Jin W, Kim DH (2018). Design and Implementation of e-Health System Based on Semantic Sensor Network Using IETF YANG. Sensors (Basel).

[67] Nachabe L, Girod-Genet M, El Hassan B (2015). Unified Data Model for Wireless Sensor Network. IEEE Sensors J.

[68] Cavalini LT, Cook TW (2015). Semantic interoperability of controlled vocabularies in medicine: A case study of the International Statistical Classification of Diseases ‘Tuberculosis’ subset. Computers in Industry.

[69] Gavrilov G, Vlahu-Gjorgievska E, Trajkovik V (2019). Healthcare data warehouse system supporting cross-border interoperability. Health Informatics J.

[70] Pathak N, Mukherjee A, Misra S (2023). SemBox: Semantic Interoperability in a Box for Wearable e-Health Devices. IEEE J. Biomed. Health Inform.

[71] Behnke M, Valik JK, Gubbels S, Teixeira D, Kristensen B, Abbas M, van Rooden SM, Gastmeier P, van Mourik MS, van Mourik MS, van Rooden SM, Abbas M, Aspevall O, Astagneau P, Bonten MJ, Carrara E, Gomila-Grange A, de Greeff SC, Gubbels S, Harrison W, Humphreys H, Johansson A, Koek MB, Kristensen B, Lepape A, Lucet J, Mookerjee S, Naucler P, Palacios-Baena ZR, Presterl E, Pujol M, Reilly J, Roberts C, Tacconelli E, Teixeira D, Tängdén T, Valik JK, Behnke M, Gastmeier P (2021). Information technology aspects of large-scale implementation of automated surveillance of healthcare-associated infections. Clinical Microbiology and Infection.

[72] Iliuta M.-E., Caramihai S. I., Pop E., Moisescu, M. A., Tiganoaia B. (2023). A Digital Twin Based Approach in Healthcare.

[73] Cernian A, Tiganoaia B, Sacala I, Pavel A, Iftemi A (2020). PatientDataChain: A Blockchain-Based Approach to Integrate Personal Health Records. Sensors.

[74] Nagasubramanian G, Sakthivel RK, Patan R, Gandomi AH, Sankayya M, Balusamy B (2018). Securing e-health records using keyless signature infrastructure blockchain technology in the cloud. Neural Comput & Applic.

[75] Schweitzer M, Flórez K, Steger B, Baumgarten D, Romano V, Augustin M (2023). Integrating a Novel Eye Imaging System into Clinical Practice: An Open-Source DICOM Simulation Platform. Stud Health Technol Inform.

[76] Rubí JNS, Gondim PRDL (2020). Interoperable Internet of Medical Things platform for e-Health applications. International Journal of Distributed Sensor Networks.

[77] S. Rubí JN, L. Gondim PR (2019). IoMT Platform for Pervasive Healthcare Data Aggregation, Processing, and Sharing Based on OneM2M and OpenEHR. Sensors.

[78] Feng H, Shi B, Cao X, Hong X, Duan X, Zhong D (2019). The Conceptual Modeling of Interoperability Framework of Heart Sound Monitor in the Context of an Interoperable End-to-End Architecture. Telemedicine and e-Health.

[79] Ouffoue, G. L. A., Zaidi F., Cavalli A. R., Lallali M. (2017). An Attack-Tolerant Framework for Web Services.

[80] Aburukba R., Sagahyroon A., Elnawawy M. (2017). Remote patient health monitoring cloud brokering services.

[81] Frontoni E, Mancini A, Baldi M, Paolanti M, Moccia S, Zingaretti P, Landro V, Misericordia P (2019). Sharing health data among general practitioners: The Nu.Sa. project. International Journal of Medical Informatics.

[82] Maia P, Baffa A., Cavalcante E., Delicato F. C., Batista T., Pires P. F. (2015). A Middleware Platform for Integrating Devices and Developing Applications in e-Health.

[83] Pereira C, Frade S., Brandao P., Correia R., Aguiar A. (2014). Integrating data and network standards into an interoperable e-Health solution. IEEE 16th International Conference on e-Health Networking, Applications and Services.

[84] Macia I (2014). Towards a semantic interoperability environment. IEEE 16th International Conference on e-Health Networking, Applications and Services.

[85] Gupta N, Gupta B (2019). Big data interoperability in e-health systems.

[86] Sfat R, Marian C. V (2022). Medical Systems Open Data Exchange Interconnection and Web Questionnaires Based on the HL7 FHIR Standards. 10th E-Health and Bioengineering Conference, EHB.

[87] Setyawan R, Hidayanto A. N., Sensuse D. I., Suryono R. R., K. Abilowo (2021). Data Integration and Interoperability Problems of HL7 FHIR Implementation and Potential Solutions: A Systematic Literature Review.

[88] Ramlrez-Ramlrez R, Coslo-Leon M., Ojeda-Carreno, D., Vazquez-Briseno M., Nieto-Hipolito J. I. (2015). Designing a gateway IEEE1451-HL7 for E-health telemonitoring services.

[89] Pahontu R, Schneider G., Bergh B., Merzweiler A. (2015). An IHE based gateway architecture to link healthcare IT with medical devices in the operating room.

[90] Vanin FNDS, Policarpo LM, Righi RDR, Heck SM, da Silva VF, Goldim J, da Costa CA (2022). A Blockchain-Based End-to-End Data Protection Model for Personal Health Records Sharing: A Fully Homomorphic Encryption Approach. Sensors.

[91] Haritha T, Anitha A (2023). Multi-Level Security in Healthcare by Integrating Lattice-Based Access Control and Blockchain- Based Smart Contracts System. IEEE Access.

[92] Ray PP, Thapa N, Dash D (2019). Implementation and Performance Analysis of Interoperable and Heterogeneous IoT-Edge Gateway for Pervasive Wellness Care. IEEE Trans. Consumer Electron.

[93] Biswas S, Sharif K, Li F, Latif Z, Kanhere SS, Mohanty SP (2020). Interoperability and Synchronization Management of Blockchain-Based Decentralized e-Health Systems. IEEE Trans. Eng. Manage.

[94] Alam S, Bhatia S, Shuaib M, Khubrani MM, Alfayez F, Malibari AA, Ahmad S (2023). An Overview of Blockchain and IoT Integration for Secure and Reliable Health Records Monitoring. Sustainability.

[95] Celdran A. H., Garcia Clemente, F. J., Weimer, and J., Lee I. (2018). ICE++: Improving security, QoS, and high availability of medical cyber-physical systems through mobile edge computing.

[96] Biswas S, Sharif K, Li F, Alam I, Mohanty SP (2022). DAAC: Digital Asset Access Control in a Unified Blockchain Based E-Health System. IEEE Trans. Big Data.

[97] Alamri B, Javed I. T., Margaria T. (2021). A GDPR-Compliant Framework for IoT-Based Personal Health Records Using Blockchain.

[98] Drogovoz V, Kubankov A. N. (2023). Conceptual Aspects of Interoperability Control for Improving Synchronization Processes in Cloud Computing. Systems of Signal Synchronization, Generating and Processing in Telecommunications.

[99] Fortino G (2018). Towards multi-layer interoperability of heterogeneous IoT platforms: The INTER-IoT approach. Internet of Things.

[100] Singh R, Baig M. M., V Sonekar S., Sawwashere S. (2023). Exploring the Potential of Distributed Ledger Technology in e-Health Monitoring.

[101] Krukowski A, Charalambides M., Chouchoulis M., Vogiatzaki E. (2014). Supporting medical research on chronic diseases using integrated health monitoring platform, in Proceedings of the 4th International Conference on Wireless Mobile Communication and Healthcare.

[102] Kumar A, Sharma, N., Chauhan R., Prabha, C., Sharma M. (2023). Blockchain in Healthcare: Types, Privacy Challenges, and Security Resilience.

[103] De Rosis S, Nuti S (2017). Public strategies for improving eHealth integration and long-term sustainability in public health care systems: Findings from an Italian case study. Int J Health Plann Mgmt.

[104] Fonseca M, Karkaletsis K., Cruz I. A., Berler A., Oliveira I. C. (2015). OpenNCP: A novel framework to foster cross-border e-Health services. Studies in Health Technology and Informatics.

[105] Rakic D (2018). Blockchain technology in healthcare. ICT4AWE.

[106] Hendrick E, Schooley, B., Gao C. (2013). CloudHealth: Developing a reliable cloud platform for healthcare applications. IEEE 10th Consumer Communications and Networking Conference.

[107] Aburukba R, Aloul F., Mahmoud A., Kamili K., Ajmal S. (2017). AutiAid: A learning mobile application for autistic children.

[108] Radwan, A. S., Abdel-Hamid, A. A., Hanafy Y. (2012). Cloud-based service for secure electronic medical record exchange.

[109] Kliem A, Boelke A, Grohnert A, Traeder N (2016). A Reconfigurable Middleware for On-demand Integration of Medical Devices. IRBM.

[110] Kliem A., Boelke A., Grohnert A., Traeder N. (2014). Self-adaptive middleware for ubiquitous medical device integration.

[111] Ni Q, Pau de la Cruz I, García Hernando AB (2016). A foundational ontology-based model for human activity representation in smart homes. AIS.

[112] Cardoso JL, de WL, Pires LF, do Prado AF (2016). A methodology based on openEHR archetypes and software agents for developing e-health applications reusing legacy systems. Comput Methods Programs Biomed.

[113] Adel E, El-Sappagh S, Barakat S, Elmogy M (2017). Distributed electronic health record based on semantic interoperability using fuzzy ontology: a survey. International Journal of Computers and Applications.

[114] AlSobeh AM, Klaib AF, AlYahya A (2019). A national framework for e-health data collection in Jordan with current practices. IJCAT.

[115] Liu Y, Shan G, Liu Y, Alghamdi A, Alam I, Biswas S (2022). Blockchain Bridges Critical National Infrastructures: E-Healthcare Data Migration Perspective. IEEE Access.

[116] Frontoni E., Baldi M., Zingaretti P., Landro V., Misericordia P. (2014). Security issues for data sharing and service interoperability in eHealth systems: The Nu.Sa. test bed.

[117] Georgi N, Corvol A, Le Bouquin Jeannes R (2018). Middleware Architecture for Health Sensors Interoperability. IEEE Access.

[118] Franz A, Krauss, O. (2015). A Comparison of Data Traffic in Standardized Personal Health Monitoring Solutions. International Journal of Electronics and Telecommunications.

[119] Adam A, Abubakar A, Mahmud M (2019). Sensor Enhanced Health Information Systems: Issues and Challenges. Int. J. Interact. Mob. Technol.

[120] Safdar Z, Farid S, Qadir M, Asghar K, Iqbal J, Hamdani FK (2020). A Novel Architecture for Internet of Things Based E-Health Systems. j med imaging hlth inform.

[121] Pathak N, Misra S, Mukherjee A, Kumar N (2021). HeDI: Healthcare Device Interoperability for IoT-Based e-Health Platforms. IEEE Internet Things J.

[122] Kliem, A, Hovestadt M., Kao O. (2012). Security and communication architecture for networked medical devices in mobility-aware ehealth environments.

[123] Lemus-Zúñiga L, Félix JM, Fides-Valero A, Benlloch-Dualde J, Martinez-Millana A (2022). A Proof-of-Concept IoT System for Remote Healthcare Based on Interoperability Standards. Sensors.

[124] Thuemmler C, Mival O, Benyon D, Buchanan W, Paulin A, Fricker S, Fiedler M, Grottland A, Magedanz T, Ispas I (2013). Norms and standards in modular medical architectures.

[125] Rahmani A, Thanigaivelan N, Gia T, Granados Jose, Negash Behailu, Liljeberg Pasi, Tenhunen Hannu (2015). Smart e-Health Gateway: Bringing intelligence to Internet-of-Things based ubiquitous healthcare systems. 12th Annual IEEE Consumer Communications and Networking Conference.

[126] Paraciani N, Tabozzi S., Di Pasquale D., Padula M., Biocca L., Lafortuna C., Maiuri F., Rudel D., Fisk M. (2017). The TeleSCoPE Code. Quality standards for telehealth practice across Europe. E-Health and Bioengineering Conference.

[127] Waterson P, Eason K, Tutt D, Dent M (2012). Using HIT to deliver integrated care for the frail elderly in the UK: current barriers and future challenges. WORK: A Journal of Prevention, Assessment & Rehabilitation.

[128] Oehm JB, Riepenhausen SL, Storck M, Dugas M, Pryss R, Varghese J (2024). Integration of Patient-Reported Outcome Data Collected Via Web Applications and Mobile Apps Into a Nation-Wide COVID-19 Research Platform Using Fast Healthcare Interoperability Resources: Development Study. J Med Internet Res.

[129] Mtey MM, Dida MA (2019). Towards interoperable e-Health system in Tanzania: analysis and evaluation of the current security trends and big data sharing dynamics. IJATEE.

[130] Noury, N, Bourquard, K., Bergognon D., Schroeder J.-B. (2012). Interoperability of communicating medical devices in telemedicine - Regulations initiatives in France.

[131] Marceglia S, Rigby M, Alonso A, Keeling D, Kubitschke L, Pozzi G (2018). DEDICATE: proposal for a conceptual framework to develop dementia-friendly integrated eCare support. BioMed Eng OnLine.

[132] Morgan, D, Zilora, S., Bogaard D. (2015). UrHL7: An HL7 parsing and manipulation library.

[133] Eom D, Lee H. (2017). A holistic approach to exploring the divided standards landscape in E-Health research.

[134] Preve N (2010). Ubiquitous Healthcare Computing with Sensor Grid Enhancement with Data Management System (SEGEDMA). J Med Syst.

[135] Singh J, Bacon JM (2014). On middleware for emerging health services. J Internet Serv Appl.

[136] Dario C, Scannapieco G, Scienza R, Carraro MG, Saccavini C, Vio E, Valongo S (2014). The Neurosurgical Telecounseling Network in the Veneto Region: 4 Years of Experience of HEALTH OPTIMUM. Telemedicine and e-Health.

[137] M. Samonte, M, Giron, J., Pantoja M., Tobias A. (2023). A Blockchain-based EHR System for Interhospital Communication and Patient Data Ownership Empowerment.

[138] Lax G (2019). A Warning on Software Interoperability in e-Health. IJMLC.

[139] Buyurgan N, Rardin R. L., Jayaraman R., Varghese V. M., Burbano A. (2011). A novel GS1 data standard adoption roadmap for healthcare providers. International Journal of Healthcare Information Systems and Informatics.

[140] Weber JH, Ho J (2020). Applying Bidirectional Transformations to the Design of Interoperable EMR Systems. J Healthc Inform Res.

[141] Brandão A (2023). Health Records Management in Trust Model Blockchain-Based,?. Lecture Notes in Networks and Systems.

[142] Rac-Albu E. M., Ciobanu, V., Rac-Albu M., Popescu N. (2016). Interoperability of medical data through e-Health service in Romania. Lecture Notes in Business Information Processing.

[143] Alkarkoukly S, Rajput A.-M. (2021). An openEHR virtual patient template for pancreatic cancer. Studies in Health Technology and Informatics.

[144] Dias, M, Sousa, R., Duarte, J., Peixoto, H., Abelha A., Machado J. (2023). Enhancing Data Science Interoperability: An Innovative System forManaging OpenEHR Structures. Communications in Computer and Information Science.

[145] Maxi K, Morocho V. (2022). Integrating Medical Information Software Using Health Level Seven and FHIR: A Case Study. Integrating Medical Information Software Using Health Level Seven and FHIR: A Case Study,? in Communications in Computer and Information Science, 2022, pp. 84?.

[146] Nachabe L, Raiyee R, Falou O., Girod-Genet M., Elhassan B. (2020). Diabetes Mobile Application as a Part of Semantic Multi-Agent System for E-Health. Middle East Conference on Biomedical Engineering.

[147] Oh H, Rizo C, Enkin M, Jadad A, Powell J, Pagliari C (2005). What Is eHealth (3): A Systematic Review of Published Definitions. J Med Internet Res.

[148] He W, Zhang JZ, Wu H, Li Wenzhuo, Shetty S (2022). A Unified Health Information System Framework for Connecting Data, People, Devices, and Systems. Journal of Global Information Management (JGIM).

[149] Oliveira D, Miranda R, Leuschner P, Abreu N, Santos MF, Abelha A, Machado J (2021). OpenEHR modeling: improving clinical records during the COVID-19 pandemic. Health Technol.

[150] Li X, Tao B, Dai H, Imran M, Wan D, Li D (2021). Is blockchain for Internet of Medical Things a panacea for COVID-19 pandemic?. Pervasive and Mobile Computing.

[151] Kaladharan S, Manayath D, Patri R (2024). Barriers to blockchain-enabled drug recycling: A TISM-MICMAC approach. Sustainable Chemistry and Pharmacy.

[152] Khan S, Khan MI, Singh R (2023). Modeling the Barriers of Blockchain Technology implementation in Supply Chain. J. Ind. Intg. Mgmt.

[153] Sargent CS, Breese JL (2023). Blockchain Barriers in Supply Chain: A Literature Review. Journal of Computer Information Systems.

[154] Prashar A, Sunder M V (2024). Blockchain barriers in hospitals: a stakeholder theoretic perspective. BIJ.

[155] Zhong D, Kirwan MJ, Duan X (2013). Regulatory Barriers Blocking Standardization of Interoperability. JMIR Mhealth Uhealth.

