# Supplementary material for: Enhancing Interoperability for a Sustainable, Patient-Centric Health Care Value Chain: Systematic Review for Taxonomy Development
Source: J Med Internet Res. 2025 Apr 25;27:e69465. doi: 10.2196/69465 (PMC12064981; doi:10.2196/69465)
Supplement: Multimedia Appendix 1 [file jmir_v27i1e69465_app1.pdf]

Section/topic	#	Checklist item	Location(s) Reported
**INFORMATION SOURCES AND METHODS**			
Database name	1	Name each individual database searched, stating the platform for each.	Methods / Search strategy / paragraph 1 ("We consulted …"
Multi-database searching	2	If databases were searched simultaneously on a single platform, state the name of the platform, listing all of the databases searched.	Not applicable
Study registries	3	List any study registries searched.	Not applicable
Online resources and browsing	4	Describe any online or print source purposefully searched or browsed (e.g., tables of contents, print conference proceedings, web sites), and how this was done.	Not applicable
Citation searching	5	Indicate whether cited references or citing references were examined, and describe any methods used for locating cited/citing references (e.g., browsing reference lists, using a citation index, setting up email alerts for references citing included studies).	Not applicable
Contacts	6	Indicate whether additional studies or data were sought by contacting authors, experts, manufacturers, or others.	Not applicable
Other methods	7	Describe any additional information sources or search methods used.	Not applicable
**SEARCH STRATEGIES**			
Full search strategies	8	Include the search strategies for each database and information source, copied and pasted exactly as run.	Methods / Search strategy / Text box 1
Limits and restrictions	9	Specify that no limits were used, or describe any limits or restrictions applied to a search (e.g., date or time period, language, study design) and provide justification for their use.	Methods / Search strategy (from 2nd paragraph until the end, including the 2 text boxes)
Search filters	10	Indicate whether published search filters were used (as originally designed or modified), and if so, cite the filter(s) used.	Methods / Search strategy (from 2nd paragraph until the end, including the 2 text boxes)
Prior work	11	Indicate when search strategies from other literature reviews were adapted or reused for a substantive part or all of the search, citing the previous review(s).	Methods / Search strategy / 3rd paragraph, regarding the timespan
Updates	12	Report the methods used to update the search(es) (e.g., rerunning searches, email alerts).	Not applicable
Dates of searches	13	For each search strategy, provide the date when the last search occurred.	Results / Data extraction / First paragraph ("The data was retrieved …"
**PEER REVIEW**			
Peer review	14	Describe any search peer review process.	Methods / Search strategy / 3rd paragraph, from "The elegibility criterion …"
**MANAGING RECORDS**			
Total Records	15	Document the total number of records identified from each database and other information sources.	Results / Data extraction / Second paragraph ("After that …")
Deduplication	16	Describe the processes and any software used to deduplicate records from multiple database searches and other information sources.	Methods / data analysis and syntesis / Third paragraph ("Duplicated documents …")

PRISMA-S: An Extension to the PRISMA Statement for Reporting Literature Searches in Systematic Reviews
Rethlefsen ML, Kirtley S, Waffenschmidt S, Ayala AP, Moher D, Page MJ, Koffel JB, PRISMA-S Group.
Last updated February 27, 2020.

Link: https://www.equator-network.org/reporting-guidelines/prisma-s/
